# Entangled Parametric Hierarchies: Problems for an Overspecified Universal Grammar

**DOI:** 10.1371/journal.pone.0072357

**Published:** 2013-09-03

**Authors:** Cedric Boeckx, Evelina Leivada

**Affiliations:** 1 ICREA, Barcelona, Spain; 2 Universitat de Barcelona, Barcelona, Spain; Utrecht University, Netherlands

## Abstract

This study addresses the feasibility of the classical notion of parameter in linguistic theory from the perspective of parametric hierarchies. A novel program-based analysis is implemented in order to show certain empirical problems related to these hierarchies. The program was developed on the basis of an enriched data base spanning 23 contemporary and 5 ancient languages. The empirical issues uncovered cast doubt on classical parametric models of language acquisition as well as on the conceptualization of an overspecified Universal Grammar that has parameters among its primitives. Pinpointing these issues leads to the proposal that (i) the (bio)logical problem of language acquisition does not amount to a process of triggering innately pre-wired values of parameters and (ii) it paves the way for viewing language, epigenetic (‘parametric’) variation as an externalization-related epiphenomenon, whose learning component may be more important than what sometimes is assumed.

## Introduction

Ever since Chomsky’s introduction of the terms ‘principle’ and ‘parameter’, Universal Grammar (UG), conceived as the initial state of the human language faculty, has been described in terms of properties with fixed values and as such invariant across languages (‘principles’), and parameters, which are initially unvalued – hence parameterizable – principles that come equipped with a finite set of possible values and that await setting on the basis of the primary linguistic data that a child is exposed to [1]. This conception of the initial state of human language faculty (FL) has since been criticized within a generativist perspective from both a conceptual [2] and an empirical point of view [3], but also outside the generativist camp [4], [5]. The present study aims to offer arguments against a parametric approach to UG from a third perspective: the nature of parametric hierarchies – inherent to the notion of parameter –, by exploiting robust findings in the field of parametric approaches to UG that articulate the relations and hierarchies between parameters in sufficient detail so as to allow us to falsify them [6].

The notion of parameter was originally meant to have some concrete theoretical substance that would go beyond merely representing points of crosslinguistic variation. Parameters were originally conceived of as clusters of grammatical properties manifested across morphosyntactic environments and not as highly specific, point-like differences (i.e. macroparameters rather than microparameters, respectively) [1]. However, when put under empirical, crosslinguistic scrutiny, the classical notion of macroparameter, albeit theoretically plausible, proved hard to maintain in the intervening 30 years. Virtually all candidates failed to retain their ‘macro’ status, since they quickly decomposed in order to account for subtler points of variation. The observation that macroparameters ‘leak’, resulting in microparameters, has led to a number of proposals that question the feasibility of the classical notion of parameters, suggesting that this concept should be abandoned (e.g., [2], [3], [5], [7]–[10]).

If one wishes to maintain the existence of parameters in this context, arguments must be made regarding their implicational structures: to retain their feasibility, parametric proposals should make available a parametric space that is organized into certain paths, forming parametric hierarchies. Put differently, the only theoretically plausible way to go about viewing variation as parametric, in the substantive sense of the term, goes through postulating the existence of hierarchically-organized parameters (e.g., as in [11], [12] or, in much greater detail, in [6]).

Our approach targets the nature of such hierarchies as these are presented in a specific pool of data that consists of a sufficient amount – sufficient to make certain calculations robust – of hierarchically-organized, interlocked parameters (i.e. parameters whose neutralization/setability depends on the setting of other parameters) and their manifestation across a variety of languages. Identifying the relevant empirical arguments against the soundness of parametric approaches to UG proceeds through implementing a novel program-based analysis to the relevant parameters given in the pool of data. The latter is presented in the work carried out by Longobardi & Guardiano ([6]; henceforth, L&G) and consists of 62 parameters that come from the nominal domain (i.e. DP parameters).

The list of the parameters L&G propose is given in table S1, appendix S1in File S1. The developed program was modelled on the basis of the L&G pool of data and customized to address issues that arise from the hierarchical organization of interlocked parameters. Two factors make the specific pool of data a unique candidate for program analysis of parametric hierarchies: First, the fact that L&G articulate in detail and across a variety of languages the status of all the input nodes (i.e. parameters on the status of which the neutralization/setability of other parameters depends). Second, they provide the parametric dependencies that define this neutralization/setability of such dependent parameters.

Despite the fact that the pursued analysis deals with this pool of data, we strongly believe that any observations drawn from this analysis regarding the nature of the relevant hierarchies and dependencies should not be read only in relation to these specific parameters or this specific functional domain. Instead, these observations are expected to have parallels in parametric hierarchies from other functional domains, because dependencies and states aside, the program does not see the linguistic status of the parameters under examination; it simply traces issues related to their existence. In other words, the program is a Java tool that tests the realization of paths in language-parameter pairings, but in this process, it does not take into account what the linguistic manifestation of a parameter is in terms of its morphophonological realization in a given language; it only reads the values of settable nodes when these appear in a given parametric dependency. For the program (the code of which is given in appendix S3 in File S1) these values correspond to Boolean literals, detached from their specific linguistic meaning, and not to linguistic phenomena. Since these parametric dependencies form hierarchies that exist in all parametric models we know of, it is highly likely that implications discussed in relation to this model would have analogues in other parametric models.

## Materials and Methods

L&G originally identify 63 DP parameters: These are binary parameters presented alongside setting states and setability relations, across 23 contemporary and 5 ancient languages, not all of them phylogenetically related. However, the material that was eventually converted into program input for the purposes of the present study consists of values for 62 DP parameters (instead of 63 that L&G identify) across 28 languages. This difference is due to the existence of two discrepancies between what is reported in L&G (and given here in table S1) and the data set that the program received as input (henceforth, program input). First, what is referred to as parameter 62 in L&G was excluded from the program input due to an inconsistency that arises between the states that the dependency shows as necessary for 62 to be able to be set and the states that some languages have when setting 62 in reality. It should also be noted that it was precisely this parameter that was left out in subsequent work that was based on this pool of data (e.g., [13]). This elimination reduces the total number of the discussed parameters from 63 to 62, and what appears as parameter 62 in the subsequent analyses corresponds to parameter 63 (± Grammaticalized Geographical Article) in table S1.

The second discrepancy refers to the dependency that gives rise to the setability of parameter 60: From the five possible ways to satisfy the dependency and reach [60set] none of them is satisfied for Modern English and Norwegian and yet both languages set parameter 60. This parameter was not excluded from the program input; instead the dependency that gives rise to its setability was modified into {51+ OR 43− OR 44− OR 45− OR 46− OR 47−}, following a suggestion by Giuseppe Longobardi (personal communication). The [A-compl] part of the dependency was not taken into account since it is not part of the pool of data. Returning to the pool of data,

“The 28 languages were chosen from the Indo-European ones with six exceptions. They are the following: Italian (It), Salentino (Sal), Spanish (Sp), French (Fr), Portuguese (Ptg), Rumanian (Rum), Latin (Lat), Classical Greek (ClG), New Testament Greek (NTG), Grico (Gri), Modern Greek (Grk), Gothic (Got), Old English (OE), Modern English (E), German (D), Norwegian (Nor), Bulgarian (Blg), Serbo-Croatian (SC), Russian (Rus), Irish (Ir), Welsh (Wel), Hebrew (Heb), Arabic (Ar), Wolof (Wo), Hungarian (Hu), Finnish (Fin), Hindi (Hi), and Basque (Bas). The basic alternative states of each parameter are encoded as ‘+’ and ‘−’ in [table S1].

[…] Within the chosen DP module, further subdomains can be distinguished: the status of various features, such as Person, Number, Gender (param. 1–6), Definiteness (roughly 7–16), Countability and related concepts (17–24), and their impact on the syntax/semantic mapping; the grammar of genitive Case (25–31); the properties of adjectival and relative modification (32–41); the position of the head noun with respect to various elements of the DP and the different kinds of movements it undergoes (42–50); the behavior of demonstratives and other determiners, and its consequences (51–55 and, in a sense, 60–6 [2]); the syntax of possessive pronouns (56–59).” (L&G: 1688)

The setting of a parameter occurs on the basis of language data, whereas setability depends on the status [+, −] of the input parameters that the dependency specifies. If a dependency is not satisfied in a language, the corresponding parameter is marked with [0], meaning that the parameter is not settable (e.g., assuming that [5set] depends on [4−], if the latter is in any other state, the former is marked with 0 which indicates that the parameter is neutralized/not settable). [?] in L&G refers to “a few empirically uncertain states” (p. 1689), most probably uncertain in the sense that their value as [NUM+] or [NUM-] is dubious and not their status as settable vs. non-settable. These uncertain stages had to be coded somehow for the program to be able to read the logical expressions that might make use of the parameter states that these [?] reflect. Since these states do not unambiguously show the target value as either [NUM+] or [NUM-], for the purposes of the program input, [?] was treated uniformly with 0 and values opposite from the target ones (e.g., assuming that [5set] depends on [4−], if the latter reads [4+] or [0] or [?], the program returns the same outcome for [5set]: False, which corresponds to non-settable).

All +/− values were assumed as presented in L&G. Whatever discrepancies are noted in the following sections between the results of the program and the settable parameters as these are depicted in table S1 are only due to (re-)calculation issues and not due to altering judgments with respect to real-language data. The dependencies were also converted into program input as presented, after being checked for consistency with the states on which they operate, which is what led to the aforementioned exclusion of what in L&G appears as parameter 62. The setting of a given parameter does not affect the setting of another, only the setability; setting is always based on language data that L&G have collected. The overall number of the dependent and the independent parameters is 46 and 16 respectively.

As more parameters/dependencies are added to the system, the hierarchies that render some parameters settable become increasingly complex as their status depends on other parameters that are also dependent and therefore further analyzable. To give a hypothetical example, if the setability of parameter 5 (i.e. [5set]) depends on [4+] and in turn [4set] depends on [1+] with 1 being an independent parameter, the hierarchy behind [5set] goes all the way until reaching [1+]. Therefore, for the purposes of the program input, dependent parameters are analyzed to the ones that give rise to their setability. If this analysis makes use of other dependent parameters, these are analyzed as well all the way down until reaching an independent parameter. Put differently, the logical expressions that the program examined reflect the full hierarchy, i.e. the hierarchy obtained once all nodes in the hierarchy are analyzed, and not only the nodes involved in the immediate setability of a given dependent parameter.

Another property of this increasing complexity is that it allows for optionality to enter the picture. Many parametric hierarchies in L&G make use of ‘OR’: Modifying the above given hypothetical example, if [5set] depends on [4+] and either [2+] or [3+] (i.e. [5set]: {[4+], [2+] OR [3+]}), this means that some parts of the dependency are optional and there are varying ways to satisfy it and reach [5set]. In this example, the ways are two: either [4+] and [2+] or [4+] and [3+]. All instances of optionality should be read as entailing *inclusive* disjunction: One of the two states is necessary to make parameter 5 settable in this example but nothing precludes the manifestation of both.

**Figure 1:**
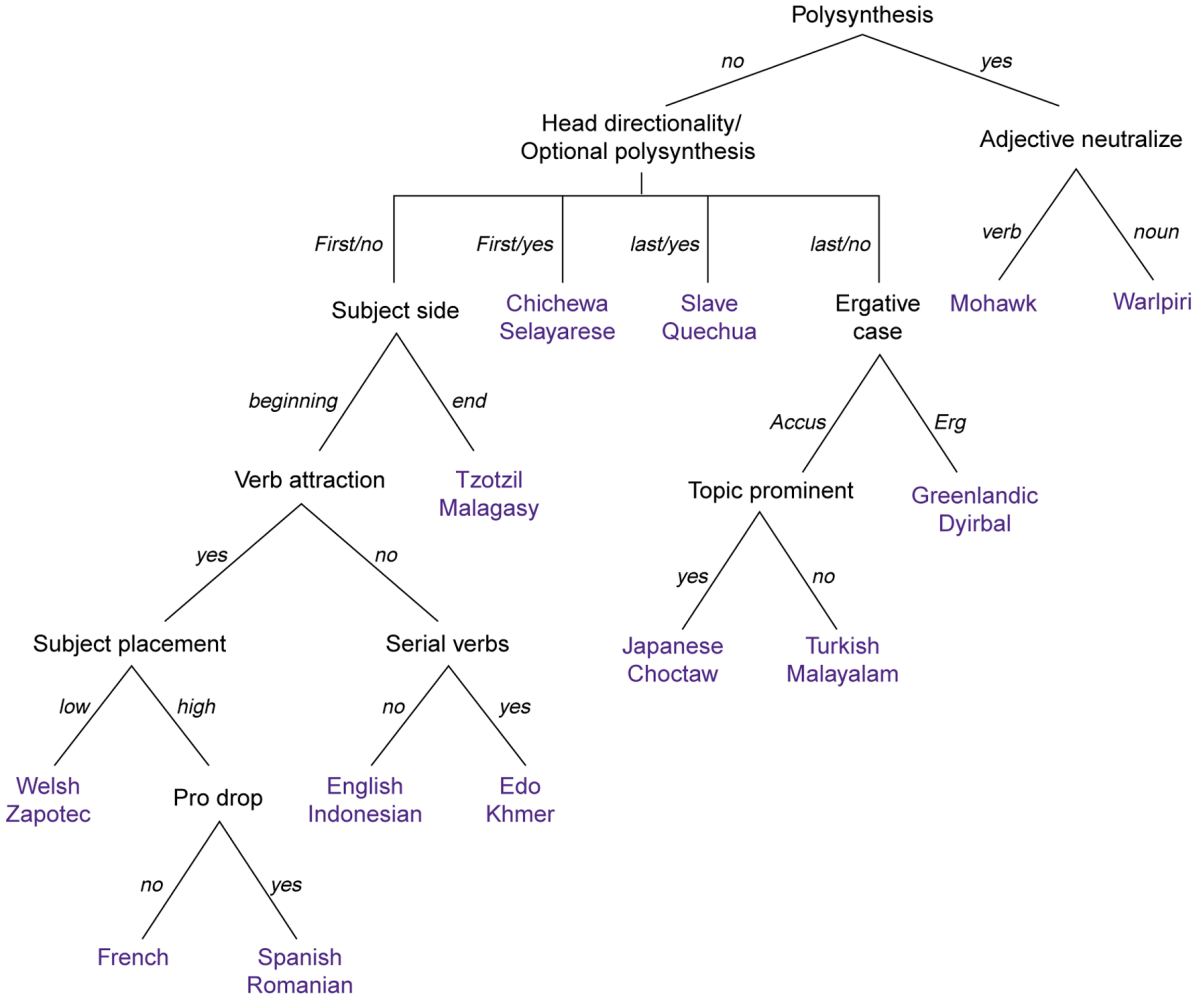
An example of parametric hierarchies [11].

Before presenting how the program-based calculation of these parametric hierarchies identifies certain empirical problems and thus offers arguments against a parametric approach to UG, it should be noted that such hierarchies are meant to organize the space of variation in a way that makes the acquisition task less burdensome [14]. As mentioned above, the notion of parameter was not intended to assume thousands of minimal points of variation as all falling within UG but instead aimed to make certain predictions with respect to the existence of specific parametric paths; for instance, along the lines of the ones presented in [11]. According to such models, UG encapsulates an ordered representation of parameters making available certain hierarchies that start off with a non-dependent parameter at the top of the hierarchy (e.g., the Polysynthesis Parameter; [15]). Obviously, these top parameters have to be set first, since their setting has an impact on the setability of the dependent parameters that follow: in Baker’s words, “an efficient learner should learn in a structured way in which some parameters are entertained first and others later” [14]. This knowledge of the “efficient learner” should be innate, given that these hierarchies are specified in UG; so not only does UG have an array of parameters and their possible values but it is further specified by flagging certain parameters as top as well as by ordering them in certain ways. This state of affairs is theoretically appealing in the sense that it reduces acquisition to a limited range of ‘set-menu’ options (e.g., as in figure 1).

**Figure 2:**
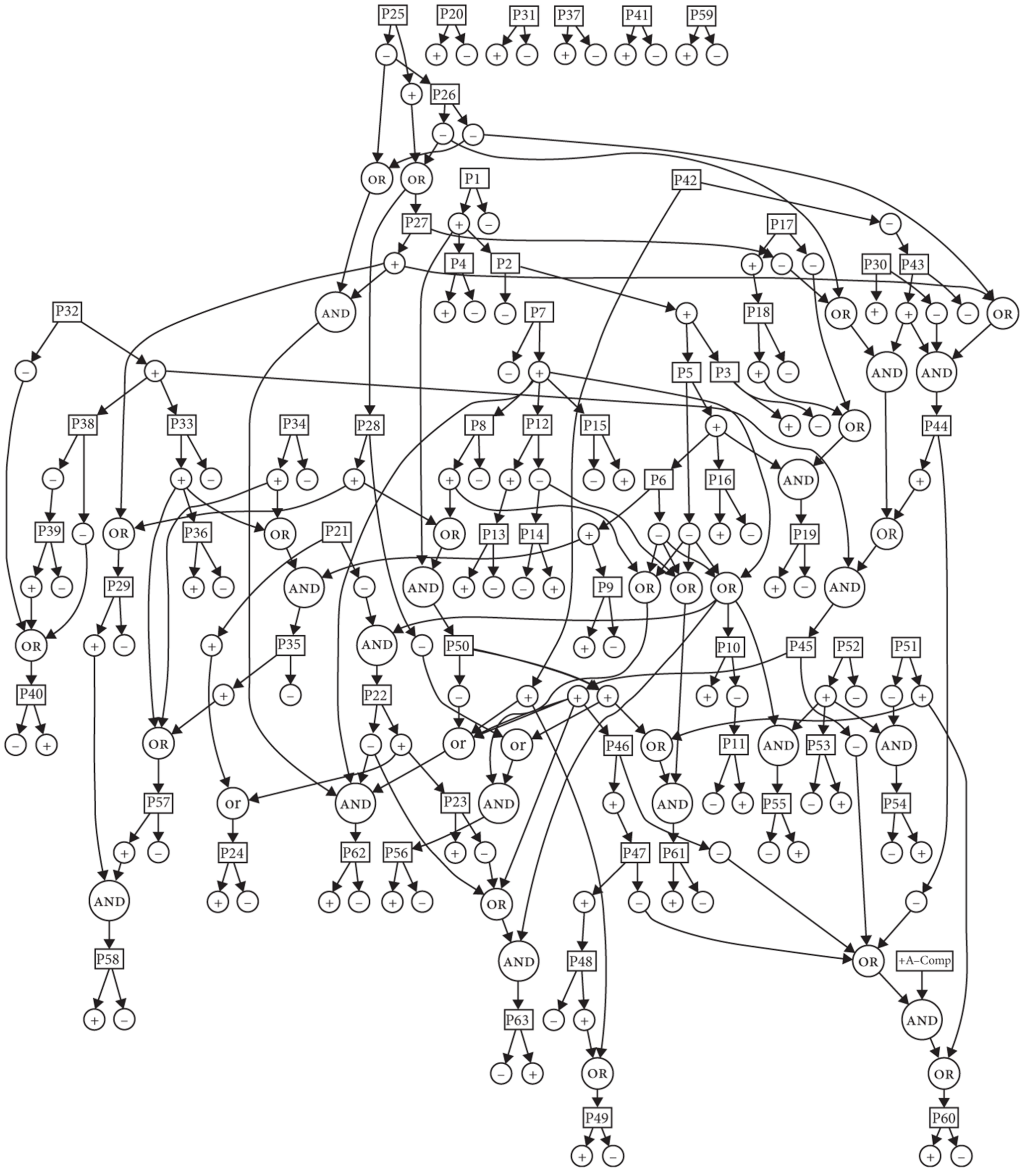
Parametric hierarchies in the nominal domain [18], [19].

Languages differ in certain ways and certain combinations have been argued to be unavailable: According to the schema in figure 1, a language cannot have both ‘verb attraction’ and ‘serial verbs’ set to ‘yes’, presumably because there is no known language manifesting both. Similarly, according to the same hierarchy, English says ‘no’ to serial verbs. However, one could suggest that some serial verb constructions still exist in English [16]. To complicate things further, where would Hebrew and Finnish be on this schema in terms of the *pro*-drop parameter? Of course, one could suggest that, since Hebrew and Finnish exhibit mixed behavior [17], *pro*-drop as a macroparameter should be articulated in more detail (i.e. microparameters in isolation) to capture the different manifestations of the parameter’s value across syntactic environments. The concern here is obvious: An overspecified UG. Yet this is not the only issue to be addressed. If one assumes subsequent parameters the setability of which is dependent on the setting of *pro*-drop, what would this mean for the representation of *pro*-drop with respect to all following parameters, as the hierarchy in figure 1 proceeds in binary fashion from top to bottom? Apparently, the theoretically appealing ‘set-menu’ parametric paths do not look as neat as figure 1 portrays them: Once more parameters and more fine-grained relationships among parameters are represented, the schema in figure 1 would progressively look more like the representation in figure 2.

The topological shift from figure 1 to figure 2 is our main concern here. Another concern pertains to crosslinguistic complexity. Assuming schematic representations that start off with a top parameter (say, polysynthesis) and then organize parameters in the ‘if *X*(yes), then *Y*; if *X*(no), then *Z*’ fashion, a child acquiring Warlpiri would have to set two parameters before reaching the end of the ‘set-menu’ option (i.e. polysynthesis to ‘yes’ and adjective neutralize to ‘noun’), whereas a child acquiring Spanish would have to set five parameters before setting *pro*-drop to ‘yes’ and reach the end of her option. In figure 1, the differences appear rather robust: there exists a 3∶1 ratio – which turns into 5∶1 if one focuses only on the dependent parameters of the schema – between the parameters that await setting in Spanish vs. Warlpiri.

One could say here that this non-trivial difference is the result of figure 1 covering a rather large amount of parametric space while not being articulated enough. If the equivalent calculations are done for the L&G pool of data, schematically represented in figure 2, one observes that the discrepancies that arise from the setability of 63 DP parameters in 28 languages are again quite wide-ranging. The maximum difference is found between Grico and Latin: 21∶10 for the dependent parameters of the network (29∶18 in the overall) with the raw numbers for settable dependent parameters being 42 and 20 (58 and 36 in the overall) for Grico and Latin respectively (see next section for details).

Observing that languages might proceed in largely dissimilar ways in terms of the number of realized nodes they involve, the question arising is how dissimilar the paths and the hierarchies that lead to setability can turn out be. This dissimilarity is not depicted in either figure 2 or table S1. However, the basis on which it can be calculated is provided. Providing insights in relation to this question can be quite revealing as to the feasibility of acquisition models that work with parameters but, more importantly, it will be revealing as to the contents of UG: The grammatical phenomena that L&G describe are meant to be understood as parameters of UG. In their words,

“grammar acquisition should reduce, for a substantial part, to parameter setting, and the core grammar of every natural language can in principle be represented by a string of binary symbols (e.g., a succession of 0,1 or +, −; cf. [20]), each coding the value of a parameter of UG” (p. 1684).

Put differently, if the proposed hierarchies are shown to run into problems once analyzed all the way down to the point of reaching the independent parameters of the hierarchy, the voiced concerns are not to be read only in relation to typology, neither are they confined to this specific pool of data, as mentioned already. Dependencies and parameter states aside, the developed program does not see the data at hand, it cannot trace the linguistic properties of the parameters under examination. It only reads logical expressions which are formed by the conjunction of Boolean literals (e.g., [7+] AND [21−]). Since logical expressions like the one given above are what all models of interlocked parameters have in common, any observed problems related to this model are highly likely to be found in all such models, once a sufficient amount of languages and parameters is built in their respective pools of data. These problems are the result of trying to capture the linguistic patterns observed across a fair amount of languages by means of parametric dependencies; they are problems *inherent* to the concept of interlocked parameters and, by extension, inherent to any theory that postulates a UG that involves interlocked parameters.

### 1. Method of Calculation

For calculating relations of setting and setability in the 28 languages at hand, any mentioned language is meant to be understood for expository purposes as whatever values the 62 parameters under discussion correspond to. Starting off from measuring nodes that await setting, the earlier mentioned calculation that showed Grico and Latin as the languages that involve the most and the least settable nodes respectively was originally done on the basis of 63 parameters. This picture does not change because the eliminated parameter is not settable to either Grico or Latin. Excluding what appears as parameter 62 in table S1, the picture that emerges for the nodes that await setting in each language is presented in table 1.

**Table 1:** Settable parameters across languages.

Languages	Overall (62)	Dependent (46)	Independent (16)
**It**	53	37	16
**Sal**	54	38	16
**Sp**	53	37	16
**Fr**	51	35	16
**Ptg**	53	37	16
**Rum**	54	38	16
**Lat**	***36***	***20***	16
**ClG**	48	32	16
**NTG**	52	36	16
**Gri**	***58***	***42***	16
**Grk**	53	37	16
**Got**	47	31	16
**OE**	53	37	16
**E**	46	30	16
**D**	50	34	16
**Nor**	49	33	16
**Blg**	52	36	16
**SC**	41	25	16
**Rus**	42	26	16
**Ir**	50	34	16
**Wel**	49	33	16
**Heb**	49	33	16
**Ar**	49	33	16
**Wo**	42	26	16
**Hu**	50	34	16
**Fin**	40	24	16
**Hi**	41	25	16
**Ba**	41	25	16

**Figure 3:**
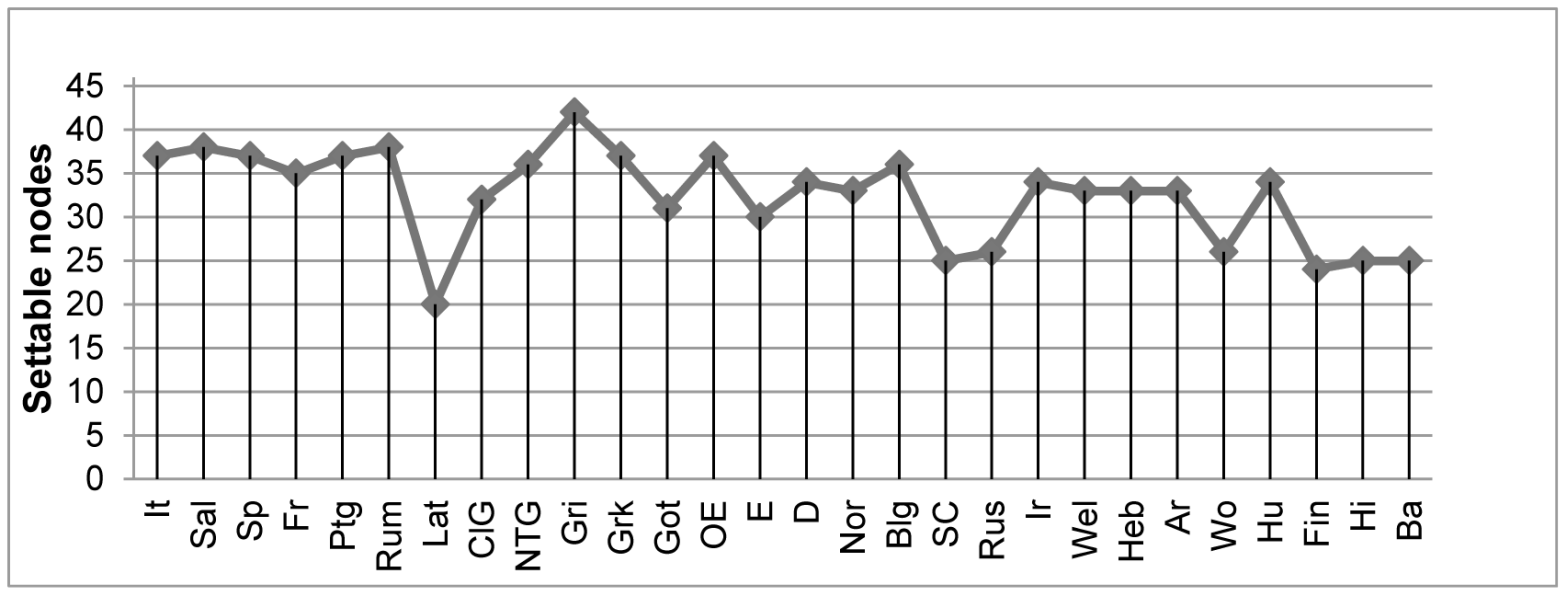
Settable dependent parameters across languages.

One should lay emphasis on the column that shows the results for dependent parameters, since this is where languages proceed in non-uniform ways. Grico and Latin lie on the edges of the continuum but they do not form the only combination showing that a significant amount of variation/non-uniformity exists in the parameter-setting task that each language requires. A schematic representation of the third column of table 1 in the form of a line chart is given in figure 3 in order to show that neither Grico nor Latin can be treated as outliers. The difference that Grico has from Salentino and Rumanian, which have 38 settable nodes, is not that robust so as to justify elimination of Grico, neither is the difference between Latin and Finnish, which has 24 settable nodes.

Even if Grico and Latin were to be excluded from the picture drawn in figure 3, the difference between Salentino and Rumanian on the one hand and Finnish on the other involves a parametric space of 14 nodes which basically amounts to more than half of the settable parametric space (in terms of dependent parameters) that Finnish has. Apparently, not only do the ‘set-menu’ options show up as far from uniform across different languages in both figure 1 and figure 2, but the degree of difference is quite large and demands an explanation since it is in sharp contrast to the species-uniform character of language acquisition. The differences here are not limited only to the notion of time. It follows from the above sketched picture that children exposed to different languages make different kinds of mistakes but, crucially, also different numbers of mistakes because of the different number of nodes they have to set. If the learning process corresponds to forming statistical hypotheses on the basis of the encountered input, it seems to be the case that the more nodes one has to formulate hypotheses for, the higher the number of erroneous hypotheses that are formulated.

If the answer to the question about the origin of these differences is the environment – in the sense that grammatical properties self-organize and change through acquisition in ways that eventually affect the quantity of the (un) explored options that a child receives as input –, there is nothing in this state of affairs that suggests a need for encoding this non-uniformity/variation in UG in the shape of ‘set-menu’ parametric paths. On the contrary, since everything hints at the role of the *environment* in deriving non-uniformity and the role of *externalization* in deriving change, it seems more plausible to tie points of variation to the factors that facilitate their very existence. In other words, it is theoretically motivated to suggest that points of variation are emergent, externalization-related by-products, rather than UG encoded options.

Figure 3 shows a significant portion of the available parametric space as an ‘unexplored area’, for the child that acquires, say, Serbo-Croatian, will never have to set 21/46 dependent parameters available in that parametric space. If, in parametric approaches to UG, UG functions like a cognitive map that pictures all the possible roads and turns that acquisition can take, at point zero the child has all the roads open and active, so there needs to be some process that renders a portion of the parametric space an ‘unexplored area’. This is the job of interlocked parameters: to organize all the roads in certain zones, in a way that if a child enters a specific zone, other zones become territories that will never be explored (in monolingual situations). This architecture, by coming in the form of (interlocked) parameters, is meant to be part of UG. In this context, figure 1 indeed looks appealing in that, leaving crosslinguistic quantitative dissimilarity of the explored nodes aside, it provides a very neat, at all levels binary, organization of zones. A key characteristic that makes figure 1 appealing is that all zones come with a single entrance, which corresponds to having only one way of reaching setability in each and every case (i.e. head directionality can be easily reconstructed into two nodes (‘first’ and ‘last’) each of which will have two branches (‘yes’ and ‘no’)). Under these assumptions, the navigation space is indeed constrained, the child will never have to consider alternative paths to setability so no extra work will be required, and the architecture of the depicted hierarchy is optimally structured.

The aim is to see whether all these theoretically appealing properties of figure 1 are retained once the variation space in question is more articulated. If they are, one may make a point in favor of the existence of a parametric UG. If they are not, the idea of the child having to consider alternative paths for setability contradicts the nature of interlocked parameters: they are supposed to constrain the space a child has to navigate, by making available certain zones, not to turn the cognitive map into a convoluted labyrinth. If it turns out that they do the latter, and given that encoding unrelated (i.e. non-interlocked) points of variation in UG is an alternative that loses the benefit of channeling variation in certain ways (i.e. hierarchies) which make acquisition less effortful in the sense that the child would have to explore certain zones but not others depending on the setting of the top-most parameters, a parameter-free version of UG emerges. Figure 2 is less optimally organized in that it brings optionality into the equation. Optimality then gives rise to multiple setability paths for the same parameter and it is not clear from figure 2 whether every dependent parameter has one way of reaching setability for each language. Similarly, it is not clear whether the different setability paths across languages are of the same complexity, with complexity here referring to the number of nodes that each path involves.

For these reasons, the setability paths of those complex parameters that allow for optionality in the L&G pool of data needed to be calculated. Table S1 lists the states of the input parameters as well as the parametric dependencies on which the setability of the dependent parameters relies, so the calculation of which and how many setability paths each language realizes is doable on the basis of the data that L&G provide. However, given the complexity of the parametric space given in figure 2, a computerized search is necessary in order to see which setability paths are available in each parameter-language pairing. In the absence of such a tool, the manual computation of all possible combinations for every language, apart from being highly time-consuming, would likely give rise to miscalculations due to the number of the states that one has to keep track of when dealing with the most complex dependencies. This is probably what justifies some of the discrepancies between the program output and what is originally listed as (non-)settable by L&G in table S1. These discrepancies are listed and explained in appendix S2 in File S1. Therefore, a program was developed and the computation was done in a semi-automatic way. Having checked manually that the dependencies are indeed respected in every case that a language sets a parameter – on this basis, original parameter 62 was excluded –, the relevant (i.e. optionality showing) portion of the dependent parameters given in table S1 was converted to program input.

**Figure 4:**
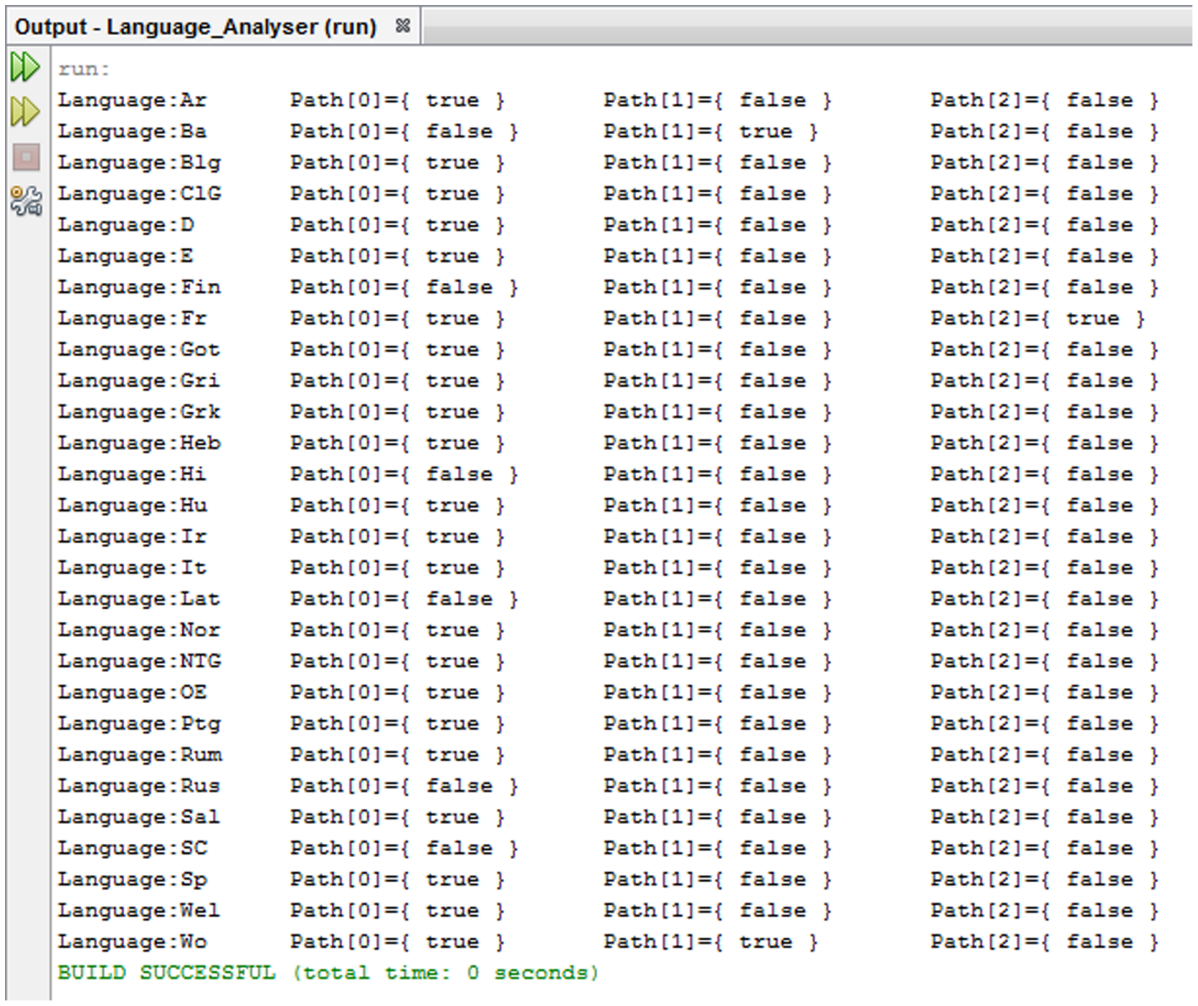
Example of program output for parameter 10.

The tool is a program implemented in Java and we used NetBeans IDE (version 7.3.1) to execute it. The code is given in appendix S3 in File S1 and figure 4 provides an example of the program output. The editable version of the code comes with instructions that make the tool user-friendly since it can be easily adapted to perform similar calculations in other pools of data of that sort. The program parses a file that contains the setability paths for each parameter-language pairing in a specific format. In this sense, it is a semi-automatic program because it takes as a prerequisite the calculation of the parametric paths (i.e. the dependencies) by the user. Paths here do not refer to the dependencies as these are given by L&G in the first column of table S1, because the dependencies that involved analyzable parameters had to be amended into a list of all the relevant nodes until reaching an independent parameter. The underlying idea is that the analysis has to proceed all the way down for the complete picture to emerge, exactly as happens in figure 1. The program output is produced as follows: Every path is converted by the user to a logical expression which is formed by the conjunction of Boolean literals. In this case, a Boolean literal is every valued parameter (e.g., [1+]) that a path makes use of in order to specify setability of another parameter. The levels of embedding were all flattened and complexity was measured in terms of the number of nodes in a path. Upon receiving the logical expressions in the form of a string of conjuncted Boolean literals, the program tested their realization in every parameter-language pairing and returned a True/False output (as shown in figure 4) for availability and non-availability of a path in a language respectively. These values are coded in appendix S2 in File S1 as 1 and 0 respectively.

## Results

It has been shown earlier that languages proceed in largely dissimilar ways in terms of the number of settable nodes they involve. What was not shown in the case of figure 1, but is shown through the program analysis for figure 2, is that languages differ yet across another dimension: the number of setability paths that each language makes available for the same parameter.

The tabularized presentation of the results in appendix S2 in File S1 can be discussed in relation to a variety of questions, such as: (i) the concept of setability (e.g., is there always one way to reach setability of a given parameter within a given language as figure 1 quite neatly suggests?), (ii) crosslinguistic uniformity (e.g., do all languages set roughly the same number of parameters or are the big differences observed in figure 1 preserved regardless of how articulated the corresponding parametric domain is?), (iii) the notion of parametric dependencies from an empirical point of view (e.g., once a sufficient number of languages and parameters is built in, do parametric dependencies end up involving mutually exclusive values within the very same path?), (iv) the tendency of languages to go for the easier rather than the most complex ways, if a dependency predicts more than one ways to reach setability of a parameter, (v) the system itself; whether it is deterministic or whether it predicts an inordinate number of setability ways that are not manifested in any language possibly due to (iv).

According to the results, not all languages have only one way to reach setability of a given parameter and, once more, crosslinguistic differences can be quite robust. Moreover, not all setability paths for a given parameter are equally complex. Quite interestingly, it turns out to be the case that the simpler paths are almost always realized, whereas most of the complex paths are not: The program output shows that languages set a complex parameter in the ‘less complex’ ways that a dependency makes available. That being said, the fact that languages typically go for the simpler setability paths that a dependency predicts may be taken to suggest that language does show some kind of optimal organization, but the concept of interlocked parameters itself may not be deterministic enough. It is not deterministic enough in the sense that once enough languages and enough dependent parameters are put into the equation, the system, by operating on combinations of increasingly complex dependencies across levels, overproduces and predicts setability paths that are not realized by any language.

To illustrate this overproduction with a hypothetical ‘toy’ example, imagine that all languages in a pool of data are able to reach setability of parameter 70 on the basis of [1+]. Assume then that an outlier is added which reaches [70set] on the basis of [23+]. Up to this point and for all the languages in this pool of data, 23 might be settable on a simple path (e.g., [22−]), but then once a second outlier is built in, 23 might be settable in a more complex way (e.g., [20+], [21+]), whereas this outlier might not set 70 at all. The system, however, by combining possible realizations of paths *across* levels, would predict as theoretically possible a setability path for 70 which would be [23+(20+, 21+)] and which would not be realized by any language from the ones that exist in the pool of data. Recall that this non-realized space is meant to be encoded in UG. Also, it would be far from a safe assumption if one argued that this space might not be realized in *these* specific languages but it will be realized if more languages are added in the system. On the contrary, it appears to be the case that when languages keep being added, the dependencies have to exponentially grow in order to capture the states that set/neutralize a complex parameter in the newly added languages. In the long run, this growth will add to the number of the complex paths, whereas languages will still not make use of the (newly-emerged) most complex paths that the system makes available.

The system might even predict as theoretically possible some setability paths that are practically impossible to realize due to conflicts in the dependency: the analysis of the setability paths for parameter 62 in appendix S2 in File S1 shows precisely this state of affairs, while the same analysis for parameter 56 is a good example to show the amount of unrealized setability paths; despite the fact that the majority of the languages at hand do set parameter 56 (i.e. realize at least one setability path and actually many of them realize not only one), more than 1/3 of the overall predicted paths remains unrealized.

## Discussion

The existence of both unrealized and unrealizable paths is a manifestation of the exact opposite effect from the one parameters were intended to have in relation to acquisition. To pursue the analogy with the ‘map’ metaphor, a cognitive map that encodes interlocked parameters is put forth as an aid to acquisition. It goes without saying that this aid is dubious if the map shows roads that are not realized in any language landscape, and this is an empirical finding that needs to be taken into account when one advocates the existence of interlocked parameters.

These observations give rise to five intimately intertwined problems that pertain to (i) cross-linguistic variability in setability relations (the *setability* problem), (ii) the (species-) uniform character of UG (the *uniformity* problem), (iii) the fixed character of the architecture of UG (the *fixity* problem), (iv) the overproduction of predicted paths by the system (the *overproduction* problem), and (iv) optimality considerations (the *optimality* problem). In their totality, these problems suggest that the notion of parametric dependencies runs into empirical problems that should cast doubt on the feasibility of parametric approaches to UG.

(i) corresponds to the *setability* problem, that is, to the fact that there is qualitative and quantitative crosslinguistic dissimilarity in terms of the setability paths that each language shows as realized. Qualitative dissimilarity boils down to varying complexity: language A might achieve setability of a parameter on the basis of a path that consists of a single node, whereas language B might achieve setability of the exact same parameter on the basis of another path that has nine nodes (this is a scenario that actually occurs for parameter 49: Arabic sets it on a single node, whereas Salentino sets it on the basis of a path that involves nine nodes). Quantitative dissimilarity boils down to optionality: language A might be able to achieve setability of a parameter on the basis of one path, whereas language B might have four paths (again, this is a scenario that actually occurs for parameter 56: French has four ways to reach setability but Basque has one). Ways to reach setability across languages should not be misunderstood as nodes that are settable across languages. Variation in the latter is fine and is the source of cross-linguistic differences in parametric models like the one in figure 1. The existence of variation in relation to the former, however, is not related to or reflected on cross-linguistic variation, in the sense that two languages might set the exact some parameters to the exact same values, but through different routes. This type of variation across but also *within* languages is something that figures 1 and 2 do not show and this variation is what complicates the neatly organized routes of variation that are traditionally illustrated with a single way of reaching dependent nodes.

The problem of crosslinguistic dissimilarity also arose when discussing setting of parameters in figure 3. The crucial difference between these two cases is that in figure 3, the problem of dissimilarity in terms of the number of parameters awaiting *setting* in each language could be remedied if one argues that the fact that the child acquiring Grico has to set more nodes than the child acquiring Finnish is the result of these two children entering different zones on the map. The problem is not remedied in the case of *setability* because varying numbers of setability paths correspond to varying numbers of entrance points on the map. Viewing the first factor as species-uniform, the uniformity of a UG architecture that has interlocked parameters is retained in the case of parameters that await setting – because the cognitive map will make available the same amount of zones across speakers of different languages –, but it is lost in the case of alternative/multiple paths of setability, because a key component of the map is shown to vary quantitatively: the number of the entrances to each zone. These entrances, which correspond to varying ways/paths of achieving setability of a parameter, eventually embroider variation on the cognitive map and this variation makes the species-uniform character of UG disappear, leading to the second problem identified above: *uniformity*.

The uniformity problem contradicts a core property of UG: one must either abandon the idea that the primitives of UG are species-uniform or give up the notion of interlocked parameters that postulates variation in terms of available setability paths. Table 2 sheds light to setability and uniformity considerations in relation to parameter 57. A cell marked with 1 in the language columns indicates that the setability conditions specified in the first column are satisfied in the respective language; hence this setability path is available in that language.

**Table 2:** Setability paths for parameter 57 (± Feature Spread on Possessives).

4 Setability Paths	It	Sal	Sp	Fr	Ptg	Rum	Lat	ClG	NTG	Gri	Grk	Got	OE	E	D	Nor	Blg	SC	Rus	Ir	Wel	Heb	Ar	Wo	Hu	Fin	Hi	Ba
34+	**1**	**1**	**1**	**1**	**1**	**1**	**1**	**1**	**1**	**1**	**1**	**1**	**1**	0	**0**	**1**	**1**	**1**	**1**	**1**	0	**1**	**1**	1	**1**	**1**	**1**	1
33+(32+)	**1**	**1**	**1**	**1**	**1**	**1**	**1**	**1**	**1**	**1**	**1**	**1**	**1**	0	**1**	**1**	**1**	**1**	**1**	**1**	0	**1**	**1**	0	**0**	**1**	**1**	0
35+(6+(5+(2+(1+))), 34+)	**1**	**1**	**1**	**0**	**1**	**1**	**1**	**1**	**1**	**1**	**1**	**1**	**1**	0	**0**	**1**	**1**	**1**	**1**	**1**	0	**1**	**1**	0	**1**	**1**	**1**	0
35+(6+(5+(2+(1+))), 33+(32+))	**1**	**1**	**1**	**0**	**1**	**1**	**1**	**1**	**1**	**1**	**1**	**1**	**1**	0	**1**	**1**	**1**	**1**	**1**	**1**	0	**1**	**1**	0	**0**	**1**	**1**	0

Table 2 illustrates that most languages that set this parameter can have it settable in four different ways. French and Hungarian have 2/4 ways, while Basque has only one. The problem of quantitative dissimilarity is not remediable here even if one argues that varying (numbers of) setability paths exist because the children that acquire Italian, French, and Basque select different options (i.e. as when they select different zones/‘set-menu’ parametric paths); the problem is that, according to table 2, they do *not* have the same pool of options to select from: the map of the one has four ways to enter [57set], whereas the maps of the others have two or one.

Point (ii) then relates the setability problem to Chomsky’s three factors in language design [21]. The first factor which refers to biological endowment (i.e. UG viewed as a cognitive map that encodes all possible variation paths through encoding parametric paths) is meant to be understood as species-uniform. Under this assumption and if the first factor is indeed species-uniform, why do the cognitive maps of acquirers of different languages show up encoding varying numbers of setability paths?

Point (iii) is the *fixity* problem: An advocate of interlocked parameters may try to save uniformity by submitting that all children do underlyingly have the same number of setability ways, but some of the ways get blocked at point zero (i.e. at the starting point of the value-setting process), depending on the zone that each child selects. This claim is ill-founded in an empirical sense because it fails to notice that the (un)availability of a setability path materializes not at the beginning but in the course of navigating the parametric space and after setting the input parameters to a target value. In other words, in table 2, the unavailability of the second setability path for reaching [57set] in Basque crystalizes not when [33set] is achieved but when 33 is not set to +. [33set] is achieved in Basque as well, so the child acquiring this language does enter a zone that has the potential to give rise to [57set], yet the second setability path is not available in Basque because parameter 33 is eventually set to −.

This empirical problem boils down to the very essence of UG as an “innate fixed nucleus” [22]. In October 1974, a debate took place between Jean Piaget and Noam Chomsky. During this debate, the nature of this “innate fixed nucleus” (i.e. UG) was subject to much discussion:

“In this sense, the final position of Piaget at Royaumont represents a manifestation of the “empiricist” position. Once the existence of a fixed nucleus is acknowledged, the contrast between the paradigms is even more remarkable. For Piaget, accounting for the stability of the fixed nucleus in terms of self-regulating mechanisms becomes the first goal of epistemology, whereas for Chomsky, the fundamental issue is precisely the specificity of the fixed nucleus and not the manner in which is fixity is attained” ([22], p.353).

Despite the fact that the two views diverge in certain ways, they converge in accepting the fixed character of UG: the issue at stake is specificity, fixity is indisputable. If one endorses this view, one cannot argue that the existence of varying numbers of setability paths (for the same parameter, across different languages) is due to the fact that certain entrances are rendered (un)available as the child navigates through the parametric space. Put differently, the *fixed* architecture of the system cannot be both fixed and moving at the same time, and yet it is moving if parts of it are continuously adjusted in the course of navigation.

(iv) and (v) are interrelated points and both are suggestive of the character of macroparametric hierarchies. The first one corresponds to the *overproduction* problem: As mentioned already, the system overproduces by predicting paths that no language, from the ones existing in the pool of data, realizes. The second point is the direct consequence of the first and it refers to the *optimality* problem: One cannot reasonably suggest that this “innate fixed nucleus” resorts to making available all these alternative setability paths within a given language. In the long run, if the setability paths multiply as new languages are taken into account and if there are 6.909 languages on the planet [23] or even more, since the calculation of this number is unclear in the absence of any non-arbitrary way of calling something a language and another thing a dialect, let alone what happens when the notion of idiolect enters the equation, UG would end up encoding an inordinate number of setability paths for a single parameter within a single language. Observing that in a sample of 62 parameters and only 28 languages, a language can show up as having five different ways to reach setability of a parameter, one can imagine first, to what an extent this number can raise if the dependency incorporates grammatical correlations found in a larger variety of languages and second, the astronomical number of all the possibilities that UG has to encapsulate, if one allocates parametric variation to it.

Another factor that adds to this issue by raising complexity considerations comes from the notion of trigger in the parameter-setting process. L&G define triggers following [20]: “A sentence σ expresses a parameter *p_i_* just in case a grammar must have *p_i_* set to some definite value in order to assign a well-formed representation to σ”. In works that discuss the nature and the different types of triggers (e.g., [24]) one sees an idealization of the learning path that relies on a “*simplifying assumption* that the learning mechanism would rely solely on globally available [i.e. fully unambiguous] triggers for any parameter that has them” (p. 97, emphasis added). What is important here is that parameter hierarchies of the type proposed in Baker’s work are linked with *conditioned* triggers (i.e. triggers whose validity and not just availability depends on the setting of one or more parameters) [24]. Pursuing this line of thinking implies that not only the setability of dependent parameters but also the validity of triggers might vary. Complexity considerations then enter the picture once more, since as soon as one has to deal with a space that encodes a sufficient amount of parameters, it becomes virtually impossible to guarantee a positive learnability path, since parameter settings will overlap in complex ways ([25], building on [26]).

In a nutshell, these five issues suggest that the classical notion of parameter, alongside the parametric hierarchies it predicts, should be revisited, possibly from the perspective of viewing linguistic diversity not as ‘syntactic’ variation that is the result of UG-encoded parametric paths, but as the by-product of learning processes. There exists a number of recent proposals that defend this view from a theoretical perspective, through supporting a constructivist or developmentalist view to the logical problem of language acquisition [27]. Under such approaches, one of the key characteristics of the efficient learner is not the need to start the learning process from setting the parameters flagged as top-most, but the ability to integrate in the process of learning conflicting tendencies, such as the need to formulate generalizations, without however making the acquisition task more burdensome, by forming assumptions that may later hard to retract from. Hierarchical Bayesian models [28] capture the notion of overhypothesis by allowing hypothesis spaces at various levels of abstraction [29].

The present work offers empirical arguments against assuming an overspecified UG, through pinpointing problems that derive from models that operate on the basis of parametric hierarchies. These problems are to be read in the context of language acquisition in the following sense: If the hierarchies that arise from interlocked parameters are shown to run into certain empirical problems, this state of affairs would be suggestive both in terms of the nature of variation (i.e. it is not UG-derived) and of UG (i.e. it does not specify parameters alongside their triggers, the range of possible values, their setability relations etc.). Then, by virtue of the latter and viewing language as an organ of the human biology, hierarchies are also suggestive with respect to the (bio)logical problem of language acquisition, which would no longer amount to a process of triggering UG-specified, prewired values of unfixed principles.

## Supporting Information

File S1 **Appendix S1:** Pool of Data. **Appendix S2:** Tabularized Setability Paths. **Appendix S3:** Code.
(DOCX)
