# Supplementary material for: Entangled Parametric Hierarchies: Problems for an Overspecified Universal Grammar
Source: PLoS One. 2013 Sep 3;8(9):e72357. doi: 10.1371/journal.pone.0072357 (PMC3760868; doi:10.1371/journal.pone.0072357)
Supplement: File S1 — Appendix S1: Pool of Data. Appendix S2: Tabularized Setability Paths. Appendix S3: Code. (DOCX) [file pone.0072357.s001.docx]

**Appendix S1: Pool of Data**

Table S1: 63 binary parameters within the DP domain (Longobardi & Guardiano 2009: 1697)


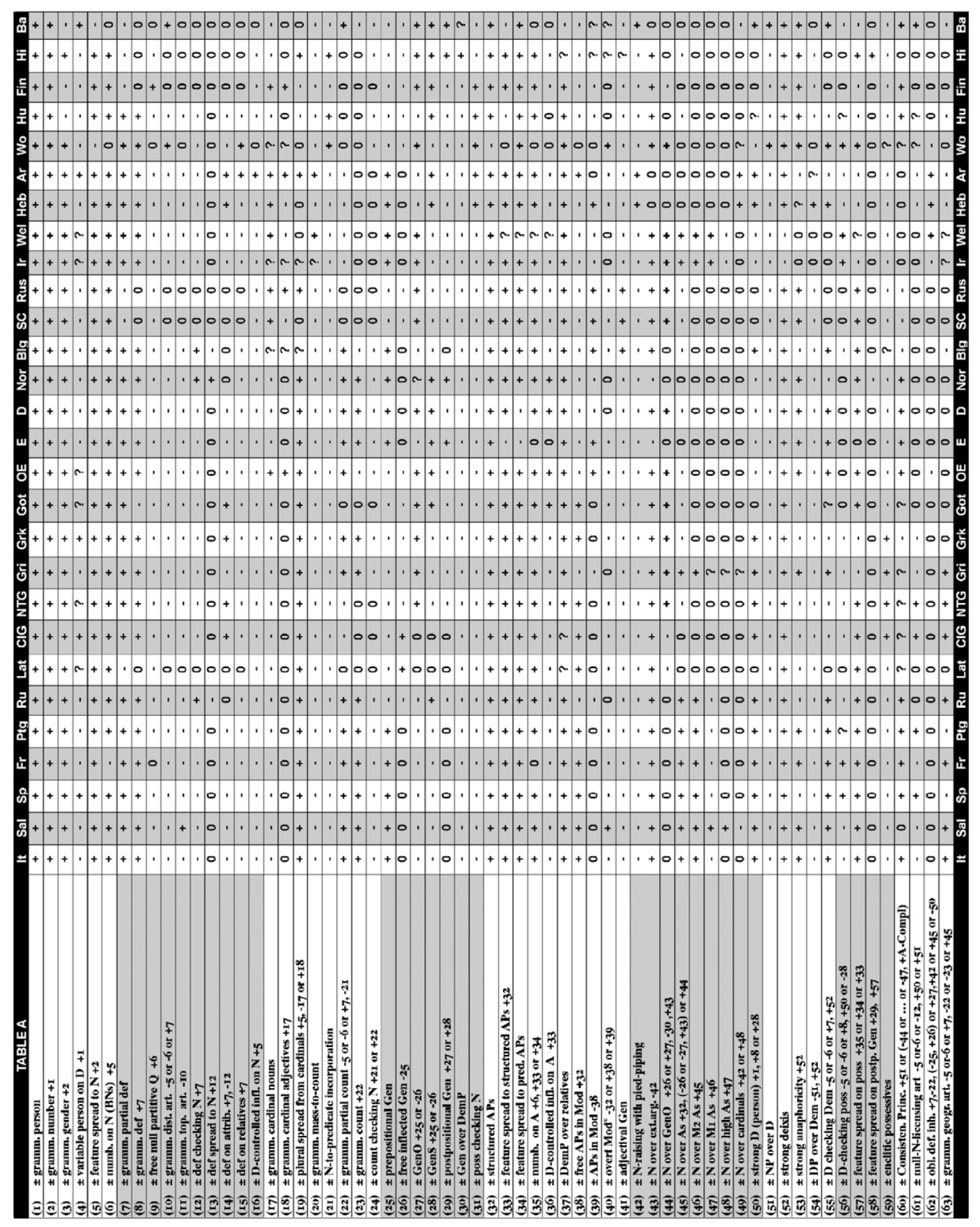


**Appendix S2: Tabularized Setability Paths**

*Reading key*: 1 signals the availability of the corresponding setability path in the relevant language, whereas 0 signals the unavailability (e.g., if a language reaches [5set] on the basis of [2+], the [2+] setability path under parameter 5 is marked with 1 for all languages that are able to set parameter 5 on the basis of having parameter 2 set to + and with 0 for all languages that have 2 in any other state: uncertain, neutralized, or set to the opposite value). When a node has an attached parenthesis to its right (e.g., 2+(1+)), the node inside the parenthesis is the analysis of the node outside the parenthesis. For example, if there is a sequence like [6-(5+(2+(1+)))], this means that [6set] depends on [5+], [5set] on [2+], [2set] on [1+] and [1] is an independent parameter. A boldfaced vertical line of markings in a language column indicates that multiple setability paths are available in the corresponding language.

The setability paths are ordered within parameters in terms of number of nodes in the dependency, starting from the least complex one. If a setability path in the first column appears in dark gray, this means that the path is not realized by any language in this pool of data (i.e. the relevant line is marked exclusively with ‘0’ for all languages). If a dependency in the first column appears in light(er) gray, this means that the path is realized in some language(s); however, in this/these language(s), a simpler path for reaching setability is also available. If mutually exclusive values are traced in a dependency, the path is marked with an asterisk. Finally, if there is a discrepancy between the program output that is reported in the following tables and what appears as set(able) in L&G (table S1), the relevant marking appears underlined and explained below the table in which it occurs.

Realization of parameter-language pairings across languages and parameters

Parameter 10: *± Grammaticalized Distal Article*

| 3 Setability Paths | **It** | **Sal** | **Sp** | **Fr** | **Ptg** | **Rum** | **Lat** | **ClG** | **NTG** | **Gri** | **Grk** | **Got** | **OE** | **E** | **D** | **Nor** | **Blg** | **SC** | **Rus** | **Ir** | **Wel** | **Heb** | **Ar** | **Wo** | **Hu** | **Fin** | **Hi** | **Ba** |
| --- | --- | --- | --- | --- | --- | --- | --- | --- | --- | --- | --- | --- | --- | --- | --- | --- | --- | --- | --- | --- | --- | --- | --- | --- | --- | --- | --- | --- |
| 7+ | 1 | 1 | 1 | **1** | 1 | 1 | 0 | 1 | 1 | 1 | 1 | 1 | 1 | 1 | 1 | 1 | 1 | 0 | 0 | 1 | 1 | 1 | 1 | **1** | 1 | 0 | 0 | 0 |
| 5-(2+(1+)) | 0 | 0 | 0 | **0** | 0 | 0 | 0 | 0 | 0 | 0 | 0 | 0 | 0 | 0 | 0 | 0 | 0 | 0 | 0 | 0 | 0 | 0 | 0 | **1** | 0 | 0 | 0 | 1 |
| 6-(5+(2+(1+))) | 0 | 0 | 0 | **1** | 0 | 0 | 0 | 0 | 0 | 0 | 0 | 0 | 0 | 0 | 0 | 0 | 0 | 0 | 0 | 0 | 0 | 0 | 0 | **0** | 0 | 0 | 0 | 0 |

Parameter 11: *± Grammaticalized Topic Article*

| 3 Setability Paths | **It** | **Sal** | **Sp** | **Fr** | **Ptg** | **Rum** | **Lat** | **ClG** | **NTG** | **Gri** | **Grk** | **Got** | **OE** | **E** | **D** | **Nor** | **Blg** | **SC** | **Rus** | **Ir** | **Wel** | **Heb** | **Ar** | **Wo** | **Hu** | **Fin** | **Hi** | **Ba** |
| --- | --- | --- | --- | --- | --- | --- | --- | --- | --- | --- | --- | --- | --- | --- | --- | --- | --- | --- | --- | --- | --- | --- | --- | --- | --- | --- | --- | --- |
| 10-(7+) | 1 | 1 | 1 | **1** | 1 | 1 | 0 | 1 | 1 | 1 | 1 | 1 | 1 | 1 | 1 | 1 | 1 | 0 | 0 | 1 | 1 | 1 | 1 | 0 | 1 | 0 | 0 | 0 |
| 10-(5-(2+(1+))) | 0 | 0 | 0 | **0** | 0 | 0 | 0 | 0 | 0 | 0 | 0 | 0 | 0 | 0 | 0 | 0 | 0 | 0 | 0 | 0 | 0 | 0 | 0 | 0 | 0 | 0 | 0 | 0 |
| 10-(6-(5+(2+(1+)))) | 0 | 0 | 0 | **1** | 0 | 0 | 0 | 0 | 0 | 0 | 0 | 0 | 0 | 0 | 0 | 0 | 0 | 0 | 0 | 0 | 0 | 0 | 0 | 0 | 0 | 0 | 0 | 0 |

Parameter 19: *± Plural Spread from Cardinals*

| 2 Setability Paths | **It** | **Sal** | **Sp** | **Fr** | **Ptg** | **Rum** | **Lat** | **ClG** | **NTG** | **Gri** | **Grk** | **Got** | **OE** | **E** | **D** | **Nor** | **Blg** | **SC** | **Rus** | **Ir** | **Wel** | **Heb** | **Ar** | **Wo** | **Hu** | **Fin** | **Hi** | **Ba** |
| --- | --- | --- | --- | --- | --- | --- | --- | --- | --- | --- | --- | --- | --- | --- | --- | --- | --- | --- | --- | --- | --- | --- | --- | --- | --- | --- | --- | --- |
| 17-, 5+(2+(1+)) | 1 | 1 | 1 | 1 | 1 | 1 | 1 | 1 | 1 | 1 | 1 | 1 | 0 | 1 | 1 | 1 | 0 | 0 | 0 | 0 | 0 | 0 | 0 | 0 | 1 | 0 | 1 | 0 |
| 18+(17+), 5+(2+(1+))) | 0 | 0 | 0 | 0 | 0 | 0 | 0 | 0 | 0 | 0 | 0 | 0 | 1 | 0 | 0 | 0 | 0 | 0 | 1 | 0 | 0 | 0 | 1 | 0 | 0 | 1 | 0 | 0 |

For Blg and Ir, the parameter is marked as uncertain in L&G and as neutralized here. These languages have parameters 17 and 18 marked with ‘?’, which is an uncertain state, treated by the program as ‘non-target’. Since a part of the dependency is not satisfied, paths are marked as unavailable.

Parameter 22: *± Grammaticalized Partial Count*

| 3 Setability Paths | **It** | **Sal** | **Sp** | **Fr** | **Ptg** | **Rum** | **Lat** | **ClG** | **NTG** | **Gri** | **Grk** | **Got** | **OE** | **E** | **D** | **Nor** | **Blg** | **SC** | **Rus** | **Ir** | **Wel** | **Heb** | **Ar** | **Wo** | **Hu** | **Fin** | **Hi** | **Ba** |
| --- | --- | --- | --- | --- | --- | --- | --- | --- | --- | --- | --- | --- | --- | --- | --- | --- | --- | --- | --- | --- | --- | --- | --- | --- | --- | --- | --- | --- |
| 7+ , 21- | 1 | 1 | 1 | **1** | 1 | 1 | 0 | 1 | 1 | 1 | 1 | 1 | 1 | 1 | 1 | 1 | 1 | 0 | 0 | 1 | 1 | 1 | 1 | 0 | 0 | 0 | 0 | 0 |
| 5-(2+(1+))), 21- | 0 | 0 | 0 | **0** | 0 | 0 | 0 | 0 | 0 | 0 | 0 | 0 | 0 | 0 | 0 | 0 | 0 | 0 | 0 | 0 | 0 | 0 | 0 | 0 | 0 | 0 | 0 | 1 |
| 6-(5+(2+(1+)))), 21- | 0 | 0 | 0 | **1** | 0 | 0 | 0 | 0 | 0 | 0 | 0 | 0 | 0 | 0 | 0 | 0 | 0 | 0 | 0 | 0 | 0 | 0 | 0 | 0 | 0 | 0 | 0 | 0 |

For Got, the parameter is marked as neutralized in L&G and as settable/non-neutralized here. It is settable on the first path, since Got is marked with [7+] and [21-] according to the pool of data in table S1.

Parameter 23: *± Grammaticalized Count*

| 3 Setability Paths | **It** | **Sal** | **Sp** | **Fr** | **Ptg** | **Rum** | **Lat** | **ClG** | **NTG** | **Gri** | **Grk** | **Got** | **OE** | **E** | **D** | **Nor** | **Blg** | **SC** | **Rus** | **Ir** | **Wel** | **Heb** | **Ar** | **Wo** | **Hu** | **Fin** | **Hi** | **Ba** |
| --- | --- | --- | --- | --- | --- | --- | --- | --- | --- | --- | --- | --- | --- | --- | --- | --- | --- | --- | --- | --- | --- | --- | --- | --- | --- | --- | --- | --- |
| 22+(7+, 21-) | 1 | 1 | 1 | **1** | 1 | 1 | 0 | 0 | 0 | 1 | 1 | 0 | 1 | 1 | 1 | 1 | 1 | 0 | 0 | 0 | 0 | 0 | 0 | 0 | 0 | 0 | 0 | 0 |
| 22+(5-(2+(1+)), 21-) | 0 | 0 | 0 | **0** | 0 | 0 | 0 | 0 | 0 | 0 | 0 | 0 | 0 | 0 | 0 | 0 | 0 | 0 | 0 | 0 | 0 | 0 | 0 | 0 | 0 | 0 | 0 | 1 |
| 22+(6-(5+(2+(1+))), 21-) | 0 | 0 | 0 | **1** | 0 | 0 | 0 | 0 | 0 | 0 | 0 | 0 | 0 | 0 | 0 | 0 | 0 | 0 | 0 | 0 | 0 | 0 | 0 | 0 | 0 | 0 | 0 | 0 |

Parameter 24: *± Count-Checking N*

| 4 Setability Paths | **It** | **Sal** | **Sp** | **Fr** | **Ptg** | **Rum** | **Lat** | **ClG** | **NTG** | **Gri** | **Grk** | **Got** | **OE** | **E** | **D** | **Nor** | **Blg** | **SC** | **Rus** | **Ir** | **Wel** | **Heb** | **Ar** | **Wo** | **Hu** | **Fin** | **Hi** | **Ba** |
| --- | --- | --- | --- | --- | --- | --- | --- | --- | --- | --- | --- | --- | --- | --- | --- | --- | --- | --- | --- | --- | --- | --- | --- | --- | --- | --- | --- | --- |
| 21+ | 0 | 0 | 0 | **0** | 0 | 0 | 0 | 0 | 0 | 0 | 0 | 0 | 0 | 0 | 0 | 0 | 0 | 0 | 0 | 0 | 0 | 0 | 0 | 1 | 1 | 0 | 1 | 0 |
| 22+(7+ , 21-) | 1 | 1 | 1 | **1** | 1 | 1 | 0 | 0 | 0 | 1 | 1 | 0 | 1 | 1 | 1 | 1 | 1 | 0 | 0 | 0 | 0 | 0 | 0 | 0 | 0 | 0 | 0 | 0 |
| 22+(5-(2+(1+))), 21-) | 0 | 0 | 0 | **0** | 0 | 0 | 0 | 0 | 0 | 0 | 0 | 0 | 0 | 0 | 0 | 0 | 0 | 0 | 0 | 0 | 0 | 0 | 0 | 0 | 0 | 0 | 0 | 1 |
| 22+(6-(5+(2+(1+)))), 21- | 0 | 0 | 0 | **1** | 0 | 0 | 0 | 0 | 0 | 0 | 0 | 0 | 0 | 0 | 0 | 0 | 0 | 0 | 0 | 0 | 0 | 0 | 0 | 0 | 0 | 0 | 0 | 0 |

Parameter 27: *± Genitive O*

| 2 Setability Paths | **It** | **Sal** | **Sp** | **Fr** | **Ptg** | **Rum** | **Lat** | **ClG** | **NTG** | **Gri** | **Grk** | **Got** | **OE** | **E** | **D** | **Nor** | **Blg** | **SC** | **Rus** | **Ir** | **Wel** | **Heb** | **Ar** | **Wo** | **Hu** | **Fin** | **Hi** | **Ba** |
| --- | --- | --- | --- | --- | --- | --- | --- | --- | --- | --- | --- | --- | --- | --- | --- | --- | --- | --- | --- | --- | --- | --- | --- | --- | --- | --- | --- | --- |
| 25+ | 1 | 1 | 1 | 1 | 1 | 0 | 0 | 0 | 0 | 0 | 0 | 0 | 0 | 1 | 1 | 1 | 1 | 0 | 0 | 1 | 1 | 1 | 1 | 0 | 0 | 0 | 0 | 0 |
| 26-(25-) | 0 | 0 | 0 | 0 | 0 | 1 | 0 | 0 | 1 | 1 | 1 | 1 | 1 | 0 | 0 | 0 | 0 | 1 | 1 | 0 | 0 | 0 | 0 | 1 | 1 | 1 | 1 | 1 |

Parameter 28: *± Genitive S*

| 2 Setability Paths | **It** | **Sal** | **Sp** | **Fr** | **Ptg** | **Rum** | **Lat** | **ClG** | **NTG** | **Gri** | **Grk** | **Got** | **OE** | **E** | **D** | **Nor** | **Blg** | **SC** | **Rus** | **Ir** | **Wel** | **Heb** | **Ar** | **Wo** | **Hu** | **Fin** | **Hi** | **Ba** |
| --- | --- | --- | --- | --- | --- | --- | --- | --- | --- | --- | --- | --- | --- | --- | --- | --- | --- | --- | --- | --- | --- | --- | --- | --- | --- | --- | --- | --- |
| 25+ | 1 | 1 | 1 | 1 | 1 | 0 | 0 | 0 | 0 | 0 | 0 | 0 | 0 | 1 | 1 | 1 | 1 | 0 | 0 | 1 | 1 | 1 | 1 | 0 | 0 | 0 | 0 | 0 |
| 26-(25-) | 0 | 0 | 0 | 0 | 0 | 1 | 0 | 0 | 1 | 1 | 1 | 1 | 1 | 0 | 0 | 0 | 0 | 1 | 1 | 0 | 0 | 0 | 0 | 1 | 1 | 1 | 1 | 1 |

Parameter 29: *± Postpositional Genitive*

| 4 Setability Paths | **It** | **Sal** | **Sp** | **Fr** | **Ptg** | **Rum** | **Lat** | **ClG** | **NTG** | **Gri** | **Grk** | **Got** | **OE** | **E** | **D** | **Nor** | **Blg** | **SC** | **Rus** | **Ir** | **Wel** | **Heb** | **Ar** | **Wo** | **Hu** | **Fin** | **Hi** | **Ba** |
| --- | --- | --- | --- | --- | --- | --- | --- | --- | --- | --- | --- | --- | --- | --- | --- | --- | --- | --- | --- | --- | --- | --- | --- | --- | --- | --- | --- | --- |
| 27+(25+) | 0 | 0 | 0 | 0 | 0 | 0 | 0 | 0 | 0 | 0 | 0 | **0** | **0** | 0 | **1** | 0 | 0 | 0 | 0 | 1 | 1 | 0 | 0 | 0 | 0 | **0** | **0** | **0** |
| 28+(25+) | 0 | 0 | 0 | 0 | 0 | 0 | 0 | 0 | 0 | 0 | 0 | **0** | **0** | 1 | **1** | 1 | 0 | 0 | 0 | 0 | 0 | 1 | 1 | 0 | 0 | **0** | **0** | **0** |
| 27+(26-(25-)) | 0 | 0 | 0 | 0 | 0 | 0 | 0 | 0 | 1 | 1 | 1 | **1** | **1** | 0 | **0** | 0 | 0 | 1 | 1 | 0 | 0 | 0 | 0 | 1 | 0 | **1** | **1** | **1** |
| 28+(26-(25-)) | 0 | 0 | 0 | 0 | 0 | 1 | 0 | 0 | 0 | 0 | 0 | **1** | **1** | 0 | **0** | 0 | 0 | 0 | 0 | 0 | 0 | 0 | 0 | 0 | 1 | **1** | **1** | **1** |

Parameter 35: *± Number on A*

| 2 Setability Paths | **It** | **Sal** | **Sp** | **Fr** | **Ptg** | **Rum** | **Lat** | **ClG** | **NTG** | **Gri** | **Grk** | **Got** | **OE** | **E** | **D** | **Nor** | **Blg** | **SC** | **Rus** | **Ir** | **Wel** | **Heb** | **Ar** | **Wo** | **Hu** | **Fin** | **Hi** | **Ba** |
| --- | --- | --- | --- | --- | --- | --- | --- | --- | --- | --- | --- | --- | --- | --- | --- | --- | --- | --- | --- | --- | --- | --- | --- | --- | --- | --- | --- | --- |
| 34+, 6+(5+(2+(1+))) | **1** | **1** | **1** | 0 | **1** | **1** | **1** | **1** | **1** | **1** | **1** | **1** | **1** | 0 | 0 | **1** | **1** | **1** | **1** | **1** | 0 | **1** | **1** | 0 | 1 | **1** | **1** | 0 |
| 33+(32+), 6+(5+(2+(1+))) | **1** | **1** | **1** | 0 | **1** | **1** | **1** | **1** | **1** | **1** | **1** | **1** | **1** | 0 | 1 | **1** | **1** | **1** | **1** | **1** | 0 | **1** | **1** | 0 | 0 | **1** | **1** | 0 |

For Wel, the parameter is marked as uncertain in L&G and as neutralized here. It is shown as neutralized because parts of the dependency (parameters 33 and 34) are uncertain for Wel.

Parameter 40: *± Overt Mod^0^*

| 3 Setability Paths | **It** | **Sal** | **Sp** | **Fr** | **Ptg** | **Rum** | **Lat** | **ClG** | **NTG** | **Gri** | **Grk** | **Got** | **OE** | **E** | **D** | **Nor** | **Blg** | **SC** | **Rus** | **Ir** | **Wel** | **Heb** | **Ar** | **Wo** | **Hu** | **Fin** | **Hi** | **Ba** |
| --- | --- | --- | --- | --- | --- | --- | --- | --- | --- | --- | --- | --- | --- | --- | --- | --- | --- | --- | --- | --- | --- | --- | --- | --- | --- | --- | --- | --- |
| 32- | 0 | 0 | 0 | 0 | 0 | 0 | 0 | 0 | 0 | 0 | 0 | 0 | 0 | 0 | 0 | 0 | 0 | 0 | 0 | 0 | 0 | 0 | 0 | 1 | 0 | 0 | 0 | 0 |
| 38+(32+) | 1 | 1 | 1 | 1 | 1 | 0 | 1 | 1 | 1 | 0 | 1 | 1 | 0 | 0 | 0 | 0 | 0 | 0 | 0 | 0 | 0 | 0 | 1 | 0 | 0 | 0 | 0 | 0 |
| 39+(38-(32+)) | 0 | 0 | 0 | 0 | 0 | 0 | 0 | 0 | 0 | 0 | 0 | 0 | 1 | 1 | 0 | 0 | 1 | 1 | 1 | 0 | 0 | 1 | 0 | 0 | 0 | 0 | 0 | 0 |

For Hi and Ba, this parameter is marked as uncertain in L&G and as neutralized here. The first two paths are unavailable because both languages set parameters 32 and 38 to the opposite values from the target ones specified by the dependency. The third path involves an uncertain state, therefore 40 is marked as neutralized for both languages.

Parameter 44: *± N over Genitive O*

| 3 Setability Paths | **It** | **Sal** | **Sp** | **Fr** | **Ptg** | **Rum** | **Lat** | **ClG** | **NTG** | **Gri** | **Grk** | **Got** | **OE** | **E** | **D** | **Nor** | **Blg** | **SC** | **Rus** | **Ir** | **Wel** | **Heb** | **Ar** | **Wo** | **Hu** | **Fin** | **Hi** | **Ba** |
| --- | --- | --- | --- | --- | --- | --- | --- | --- | --- | --- | --- | --- | --- | --- | --- | --- | --- | --- | --- | --- | --- | --- | --- | --- | --- | --- | --- | --- |
| 30-, 43+(42-), 26+(25-) | 0 | 0 | 0 | 0 | 0 | 0 | 1 | 1 | 0 | 0 | 0 | 0 | 0 | 0 | 0 | 0 | 0 | 0 | 0 | 0 | 0 | 0 | 0 | 0 | 0 | 0 | 0 | 0 |
| 30-, 43+(42-), 27+(25+) | 0 | 0 | 0 | 0 | 0 | 0 | 0 | 0 | 0 | 0 | 0 | 0 | 0 | 0 | 1 | 0 | 0 | 0 | 0 | 1 | 1 | 0 | 0 | 0 | 0 | 0 | 0 | 0 |
| 30-, 43+(42-), 27+(26-(25-)) | 0 | 0 | 0 | 0 | 0 | 0 | 0 | 0 | 1 | 1 | 1 | 1 | 1 | 0 | 0 | 0 | 0 | 1 | 1 | 0 | 0 | 0 | 0 | 1 | 0 | 1 | 0 | 0 |

Parameter 45: *± N over Adjectives*

| 6 Setability Paths | **It** | **Sal** | **Sp** | **Fr** | **Ptg** | **Rum** | **Lat** | **ClG** | **NTG** | **Gri** | **Grk** | **Got** | **OE** | **E** | **D** | **Nor** | **Blg** | **SC** | **Rus** | **Ir** | **Wel** | **Heb** | **Ar** | **Wo** | **Hu** | **Fin** | **Hi** | **Ba** |
| --- | --- | --- | --- | --- | --- | --- | --- | --- | --- | --- | --- | --- | --- | --- | --- | --- | --- | --- | --- | --- | --- | --- | --- | --- | --- | --- | --- | --- |
| 32+, 43+(42-), 26-(25-) | 0 | 0 | 0 | 0 | 0 | **1** | 0 | 0 | **1** | **1** | **1** | **1** | **1** | 0 | 0 | 0 | 0 | **1** | **1** | 0 | 0 | 0 | 0 | 0 | **1** | 1 | 1 | 0 |
| 32+, 43+(42-), 27-(25+) | 1 | 1 | 1 | 1 | 1 | **0** | 0 | 0 | **0** | **0** | **0** | **0** | **0** | 0 | 0 | 0 | 1 | **0** | **0** | 0 | 0 | 0 | 0 | 0 | **0** | 0 | 0 | 0 |
| 32+, 43+(42-), 27-(26-(25-)) | 0 | 0 | 0 | 0 | 0 | **1** | 0 | 0 | **0** | **0** | **0** | **0** | **0** | 0 | 0 | 0 | 0 | **0** | **0** | 0 | 0 | 0 | 0 | 0 | **1** | 0 | 0 | 0 |
| 32+, 44+(30-, 43+(42-), 26+(25-)) | 0 | 0 | 0 | 0 | 0 | **0** | 0 | 0 | **0** | **0** | **0** | **0** | **0** | 0 | 0 | 0 | 0 | **0** | **0** | 0 | 0 | 0 | 0 | 0 | **0** | 0 | 0 | 0 |
| 32+, 44+(30-, 43+(42-), 27+(25+)) | 0 | 0 | 0 | 0 | 0 | **0** | 0 | 0 | **0** | **0** | **0** | **0** | **0** | 0 | 1 | 0 | 0 | **0** | **0** | 1 | 1 | 0 | 0 | 0 | **0** | 0 | 0 | 0 |
| 32+, 44+(30-, 43+(42-), 27+(26-(25-))) | 0 | 0 | 0 | 0 | 0 | **0** | 0 | 0 | **1** | **1** | **1** | **1** | **1** | 0 | 0 | 0 | 0 | **1** | **1** | 0 | 0 | 0 | 0 | 0 | **0** | 0 | 0 | 0 |

For Fin, this parameter is marked as neutralized in L&G and as settable/non-neutralized here. It is settable on the first path, since Fin sets to target all the relevant values.

Parameter 46: *± N over Manner 2 Adjectives*

| 6 Setability Paths | **It** | **Sal** | **Sp** | **Fr** | **Ptg** | **Rum** | **Lat** | **ClG** | **NTG** | **Gri** | **Grk** | **Got** | **OE** | **E** | **D** | **Nor** | **Blg** | **SC** | **Rus** | **Ir** | **Wel** | **Heb** | **Ar** | **Wo** | **Hu** | **Fin** | **Hi** | **Ba** |
| --- | --- | --- | --- | --- | --- | --- | --- | --- | --- | --- | --- | --- | --- | --- | --- | --- | --- | --- | --- | --- | --- | --- | --- | --- | --- | --- | --- | --- |
| 45+(32+, 43+(42-), 26-(25-)) | 0 | 0 | 0 | 0 | 0 | **1** | 0 | 0 | 0 | **1** | 0 | 0 | 0 | 0 | 0 | 0 | 0 | 0 | 0 | 0 | 0 | 0 | 0 | 0 | 0 | 0 | 0 | 0 |
| 45+(32+, 43+(42-), 27-(25+)) | 1 | 1 | 1 | 1 | 1 | **0** | 0 | 0 | 0 | **0** | 0 | 0 | 0 | 0 | 0 | 0 | 0 | 0 | 0 | 0 | 0 | 0 | 0 | 0 | 0 | 0 | 0 | 0 |
| 45+(32+, 43+(42-), 27-(26-(25-))) | 0 | 0 | 0 | 0 | 0 | **1** | 0 | 0 | 0 | **0** | 0 | 0 | 0 | 0 | 0 | 0 | 0 | 0 | 0 | 0 | 0 | 0 | 0 | 0 | 0 | 0 | 0 | 0 |
| 45+(32+, 44+(30-, 43+(42-), 26+(25-))) | 0 | 0 | 0 | 0 | 0 | **0** | 0 | 0 | 0 | **0** | 0 | 0 | 0 | 0 | 0 | 0 | 0 | 0 | 0 | 0 | 0 | 0 | 0 | 0 | 0 | 0 | 0 | 0 |
| 45+(32+, 44+(30-, 43+(42-), 27+(25+))) | 0 | 0 | 0 | 0 | 0 | **0** | 0 | 0 | 0 | **0** | 0 | 0 | 0 | 0 | 0 | 0 | 0 | 0 | 0 | 1 | 1 | 0 | 0 | 0 | 0 | 0 | 0 | 0 |
| 45+(32+, 44+(30-, 43+(42-), 27+(26-(25-)))) | 0 | 0 | 0 | 0 | 0 | **0** | 0 | 0 | 0 | **1** | 0 | 0 | 0 | 0 | 0 | 0 | 0 | 0 | 0 | 0 | 0 | 0 | 0 | 0 | 0 | 0 | 0 | 0 |

Parameter 47: *± N over Manner 1 Adjectives*

| 6 Setability Paths | **It** | **Sal** | **Sp** | **Fr** | **Ptg** | **Rum** | **Lat** | **ClG** | **NTG** | **Gri** | **Grk** | **Got** | **OE** | **E** | **D** | **Nor** | **Blg** | **SC** | **Rus** | **Ir** | **Wel** | **Heb** | **Ar** | **Wo** | **Hu** | **Fin** | **Hi** | **Ba** |
| --- | --- | --- | --- | --- | --- | --- | --- | --- | --- | --- | --- | --- | --- | --- | --- | --- | --- | --- | --- | --- | --- | --- | --- | --- | --- | --- | --- | --- |
| 46+(45+(32+, 43+(42-), 26-(25-))) | 0 | 0 | 0 | 0 | 0 | **1** | 0 | 0 | 0 | **1** | 0 | 0 | 0 | 0 | 0 | 0 | 0 | 0 | 0 | 0 | 0 | 0 | 0 | 0 | 0 | 0 | 0 | 0 |
| 46+(45+(32+, 43+(42-), 27-(25+))) | 1 | 1 | 1 | 1 | 1 | **0** | 0 | 0 | 0 | **0** | 0 | 0 | 0 | 0 | 0 | 0 | 0 | 0 | 0 | 0 | 0 | 0 | 0 | 0 | 0 | 0 | 0 | 0 |
| 46+(45+(32+, 43+(42-), 27-(26-(25-)))) | 0 | 0 | 0 | 0 | 0 | **1** | 0 | 0 | 0 | **0** | 0 | 0 | 0 | 0 | 0 | 0 | 0 | 0 | 0 | 0 | 0 | 0 | 0 | 0 | 0 | 0 | 0 | 0 |
| 46+(45+(32+, 44+(30-, 43+(42-), 26+(25-)))) | 0 | 0 | 0 | 0 | 0 | **0** | 0 | 0 | 0 | **0** | 0 | 0 | 0 | 0 | 0 | 0 | 0 | 0 | 0 | 0 | 0 | 0 | 0 | 0 | 0 | 0 | 0 | 0 |
| 46+(45+(32+, 44+(30-, 43+(42-), 27+(25+)))) | 0 | 0 | 0 | 0 | 0 | **0** | 0 | 0 | 0 | **0** | 0 | 0 | 0 | 0 | 0 | 0 | 0 | 0 | 0 | 1 | 1 | 0 | 0 | 0 | 0 | 0 | 0 | 0 |
| 46+(45+(32+, 44+(30-, 43+(42-), 27+(26-(25-))))) | 0 | 0 | 0 | 0 | 0 | **0** | 0 | 0 | 0 | **1** | 0 | 0 | 0 | 0 | 0 | 0 | 0 | 0 | 0 | 0 | 0 | 0 | 0 | 0 | 0 | 0 | 0 | 0 |

Parameter 48: *± N over High Adjectives*

| 6 Setability Paths | **It** | **Sal** | **Sp** | **Fr** | **Ptg** | **Rum** | **Lat** | **ClG** | **NTG** | **Gri** | **Grk** | **Got** | **OE** | **E** | **D** | **Nor** | **Blg** | **SC** | **Rus** | **Ir** | **Wel** | **Heb** | **Ar** | **Wo** | **Hu** | **Fin** | **Hi** | **Ba** |
| --- | --- | --- | --- | --- | --- | --- | --- | --- | --- | --- | --- | --- | --- | --- | --- | --- | --- | --- | --- | --- | --- | --- | --- | --- | --- | --- | --- | --- |
| 47+(46+(45+(32+, 43+(42-), 27-(25+)))) | 0 | 1 | 0 | 0 | 0 | 0 | 0 | 0 | 0 | 0 | 0 | 0 | 0 | 0 | 0 | 0 | 0 | 0 | 0 | 0 | 0 | 0 | 0 | 0 | 0 | 0 | 0 | 0 |
| 47+(46+(45+(32+, 43+(42-), 26-(25-)))) | 0 | 0 | 0 | 0 | 0 | 0 | 0 | 0 | 0 | 0 | 0 | 0 | 0 | 0 | 0 | 0 | 0 | 0 | 0 | 0 | 0 | 0 | 0 | 0 | 0 | 0 | 0 | 0 |
| 47+(46+(45+(32+, 43+(42-), 27-(26-(25-))))) | 0 | 0 | 0 | 0 | 0 | 0 | 0 | 0 | 0 | 0 | 0 | 0 | 0 | 0 | 0 | 0 | 0 | 0 | 0 | 0 | 0 | 0 | 0 | 0 | 0 | 0 | 0 | 0 |
| 47+(46+(45+(32+, 44+(30-, 43+(42-), 26+(25-))))) | 0 | 0 | 0 | 0 | 0 | 0 | 0 | 0 | 0 | 0 | 0 | 0 | 0 | 0 | 0 | 0 | 0 | 0 | 0 | 0 | 0 | 0 | 0 | 0 | 0 | 0 | 0 | 0 |
| 47+(46+(45+(32+, 44+(30-, 43+(42-), 27+(25+))))) | 0 | 0 | 0 | 0 | 0 | 0 | 0 | 0 | 0 | 0 | 0 | 0 | 0 | 0 | 0 | 0 | 0 | 0 | 0 | 1 | 1 | 0 | 0 | 0 | 0 | 0 | 0 | 0 |
| 47+(46+(45+(32+, 44+(30-, 43+(42-), 27+(26-(25-)))))) | 0 | 0 | 0 | 0 | 0 | 0 | 0 | 0 | 0 | 0 | 0 | 0 | 0 | 0 | 0 | 0 | 0 | 0 | 0 | 0 | 0 | 0 | 0 | 0 | 0 | 0 | 0 | 0 |

For Gri, this parameter is marked as uncertain in L&G and as neutralized here. It is shown as neutralized because the dependency involves [47+] in all paths and Gri has 47 marked as uncertain.

Parameter 49: *±N over Cardinals*

| 7 Setability Paths | **It** | **Sal** | **Sp** | **Fr** | **Ptg** | **Rum** | **Lat** | **ClG** | **NTG** | **Gri** | **Grk** | **Got** | **OE** | **E** | **D** | **Nor** | **Blg** | **SC** | **Rus** | **Ir** | **Wel** | **Heb** | **Ar** | **Wo** | **Hu** | **Fin** | **Hi** | **Ba** |
| --- | --- | --- | --- | --- | --- | --- | --- | --- | --- | --- | --- | --- | --- | --- | --- | --- | --- | --- | --- | --- | --- | --- | --- | --- | --- | --- | --- | --- |
| 42+ | 0 | 0 | 0 | 0 | 0 | 0 | 0 | 0 | 0 | 0 | 0 | 0 | 0 | 0 | 0 | 0 | 0 | 0 | 0 | 0 | 0 | 1 | 1 | 0 | 0 | 0 | 0 | 1 |
| 48+(47+(46+(45+(32+, 43+(42-), 27-(25+))))) | 0 | 1 | 0 | 0 | 0 | 0 | 0 | 0 | 0 | 0 | 0 | 0 | 0 | 0 | 0 | 0 | 0 | 0 | 0 | 0 | 0 | 0 | 0 | 0 | 0 | 0 | 0 | 0 |
| 48+(47+(46+(45+(32+, 43+(42-), 26-(25-))))) | 0 | 0 | 0 | 0 | 0 | 0 | 0 | 0 | 0 | 0 | 0 | 0 | 0 | 0 | 0 | 0 | 0 | 0 | 0 | 0 | 0 | 0 | 0 | 0 | 0 | 0 | 0 | 0 |
| 48+(47+(46+(45+(32+, 43+(42-), 27-(26-(25-)))))) | 0 | 0 | 0 | 0 | 0 | 0 | 0 | 0 | 0 | 0 | 0 | 0 | 0 | 0 | 0 | 0 | 0 | 0 | 0 | 0 | 0 | 0 | 0 | 0 | 0 | 0 | 0 | 0 |
| 48+(47+(46+(45+(32+, 44+(30-, 43+(42-), 26+(25-)))))) | 0 | 0 | 0 | 0 | 0 | 0 | 0 | 0 | 0 | 0 | 0 | 0 | 0 | 0 | 0 | 0 | 0 | 0 | 0 | 0 | 0 | 0 | 0 | 0 | 0 | 0 | 0 | 0 |
| 48+(47+(46+(45+(32+, 44+(30-, 43+(42-), 27+(25+)))))) | 0 | 0 | 0 | 0 | 0 | 0 | 0 | 0 | 0 | 0 | 0 | 0 | 0 | 0 | 0 | 0 | 0 | 0 | 0 | 0 | 0 | 0 | 0 | 0 | 0 | 0 | 0 | 0 |
| 48+(47+(46+(45+(32+, 44+(30-, 43+(42-), 27+(26-(25-))))))) | 0 | 0 | 0 | 0 | 0 | 0 | 0 | 0 | 0 | 0 | 0 | 0 | 0 | 0 | 0 | 0 | 0 | 0 | 0 | 0 | 0 | 0 | 0 | 0 | 0 | 0 | 0 | 0 |

For Wo, this parameter is marked as uncertain in L&G and as neutralized here. It is neutralized because Wo has 48 marked as neutralized and sets 42 to the opposite value than the one required by the dependency, so none of the paths is available. For Gri, this parameter is marked as uncertain in L&G and as neutralized here. It is shown as neutralized because Gri shows 48 as uncertain, whereas 42 that is required in the first path is set to the opposite value.

Parameter 50: *±Strong D (Person)*

| 3 Setability Paths | **It** | **Sal** | **Sp** | **Fr** | **Ptg** | **Rum** | **Lat** | **ClG** | **NTG** | **Gri** | **Grk** | **Got** | **OE** | **E** | **D** | **Nor** | **Blg** | **SC** | **Rus** | **Ir** | **Wel** | **Heb** | **Ar** | **Wo** | **Hu** | **Fin** | **Hi** | **Ba** |
| --- | --- | --- | --- | --- | --- | --- | --- | --- | --- | --- | --- | --- | --- | --- | --- | --- | --- | --- | --- | --- | --- | --- | --- | --- | --- | --- | --- | --- |
| 1+, 8+(7+) | 1 | 1 | 1 | 1 | 1 | **1** | 0 | 1 | 1 | 1 | 1 | 0 | **1** | **1** | **1** | **1** | 1 | 0 | 0 | 1 | 1 | **1** | **1** | 1 | **1** | 0 | 0 | 0 |
| 1+, 28+(25+) | 0 | 0 | 0 | 0 | 0 | **0** | 0 | 0 | 0 | 0 | 0 | 0 | **0** | **1** | **1** | **1** | 0 | 0 | 0 | 0 | 0 | **1** | **1** | 0 | **0** | 0 | 0 | 0 |
| 1+, 28+(26-(25-)) | 0 | 0 | 0 | 0 | 0 | **1** | 0 | 0 | 0 | 0 | 0 | 1 | **1** | **0** | **0** | **0** | 0 | 0 | 0 | 0 | 0 | **0** | **0** | 0 | **1** | 1 | 1 | 1 |

For Fin, Hi, and Got, this parameter is marked as neutralized in L&G and as settable/non-neutralized here. It is settable on the third path in all three cases, since Fin, Hi, and Got set to target all the relevant values.

Parameter 55: *±D-Checking Demonstratives*

| 3 Setability Paths | **It** | **Sal** | **Sp** | **Fr** | **Ptg** | **Rum** | **Lat** | **ClG** | **NTG** | **Gri** | **Grk** | **Got** | **OE** | **E** | **D** | **Nor** | **Blg** | **SC** | **Rus** | **Ir** | **Wel** | **Heb** | **Ar** | **Wo** | **Hu** | **Fin** | **Hi** | **Ba** |
| --- | --- | --- | --- | --- | --- | --- | --- | --- | --- | --- | --- | --- | --- | --- | --- | --- | --- | --- | --- | --- | --- | --- | --- | --- | --- | --- | --- | --- |
| 52+, 7+ | 1 | 1 | 1 | **1** | 1 | 1 | 0 | 1 | 1 | 1 | 1 | 1 | 1 | 1 | 1 | 1 | 1 | 0 | 0 | 0 | 0 | 1 | 1 | **1** | 1 | 0 | 0 | 0 |
| 52+, 5-(2+(1+)) | 0 | 0 | 0 | **0** | 0 | 0 | 0 | 0 | 0 | 0 | 0 | 0 | 0 | 0 | 0 | 0 | 0 | 0 | 0 | 0 | 0 | 0 | 0 | **1** | 0 | 0 | 0 | 1 |
| 52+, 6-(5+(2+(1+))) | 0 | 0 | 0 | **1** | 0 | 0 | 0 | 0 | 0 | 0 | 0 | 0 | 0 | 0 | 0 | 0 | 0 | 0 | 0 | 0 | 0 | 0 | 0 | **0** | 0 | 0 | 0 | 0 |

Parameter 56: *±D-Checking Possessives*

| 15 Setability Paths | **It** | **Sal** | **Sp** | **Fr** | **Ptg** | **Rum** | **Lat** | **ClG** | **NTG** | **Gri** | **Grk** | **Got** | **OE** | **E** | **D** | **Nor** | **Blg** | **SC** | **Rus** | **Ir** | **Wel** | **Heb** | **Ar** | **Wo** | **Hu** | **Fin** | **Hi** | **Ba** |
| --- | --- | --- | --- | --- | --- | --- | --- | --- | --- | --- | --- | --- | --- | --- | --- | --- | --- | --- | --- | --- | --- | --- | --- | --- | --- | --- | --- | --- |
| 8+(7+), 28-(25+) | **1** | **1** | **1** | **1** | **1** | **0** | 0 | 0 | **0** | **0** | **0** | 0 | 0 | 0 | 0 | 0 | **1** | 0 | 0 | 1 | 1 | **0** | **0** | **0** | 0 | 0 | 0 | 0 |
| 5-(2+(1+)), 28-(25+) | **0** | **0** | **0** | **0** | **0** | **0** | 0 | 0 | **0** | **0** | **0** | 0 | 0 | 0 | 0 | 0 | **0** | 0 | 0 | 0 | 0 | **0** | **0** | **0** | 0 | 0 | 0 | 0 |
| 8+(7+), 28-(26-(25-)) | **0** | **0** | **0** | **0** | **0** | **0** | 0 | 0 | **1** | **1** | **1** | 0 | 0 | 0 | 0 | 0 | **0** | 0 | 0 | 0 | 0 | **0** | **0** | **1** | 0 | 0 | 0 | 0 |
| 8+(7+), 50+(1+, 8+(7+)) | **1** | **1** | **1** | **1** | **1** | **1** | 0 | 1 | **1** | **1** | **1** | 0 | 0 | 0 | 0 | 0 | **1** | 0 | 0 | 0 | 0 | **1** | **1** | **0** | 0 | 0 | 0 | 0 |
| 5-(2+(1+)), 28-(26-(25-)) | **0** | **0** | **0** | **0** | **0** | **0** | 0 | 0 | **0** | **0** | **0** | 0 | 0 | 0 | 0 | 0 | **0** | 0 | 0 | 0 | 0 | **0** | **0** | **1** | 0 | 0 | 0 | 0 |
| 6-(5+(2+(1+))), 28-(25+) | **0** | **0** | **0** | **1** | **0** | **0** | 0 | 0 | **0** | **0** | **0** | 0 | 0 | 0 | 0 | 0 | **0** | 0 | 0 | 0 | 0 | **0** | **0** | **0** | 0 | 0 | 0 | 0 |
| 8+(7+), 50+(1+, 28+(25+)) | **0** | **0** | **0** | **0** | **0** | **0** | 0 | 0 | **0** | **0** | **0** | 0 | 0 | 0 | 0 | 0 | **0** | 0 | 0 | 0 | 0 | **1** | **1** | **0** | 0 | 0 | 0 | 0 |
| 5-(2+(1+)), 50+(1+, 8+(7+)) | **0** | **0** | **0** | **0** | **0** | **0** | 0 | 0 | **0** | **0** | **0** | 0 | 0 | 0 | 0 | 0 | **0** | 0 | 0 | 0 | 0 | **0** | **0** | **0** | 0 | 0 | 0 | 0 |
| 6-(5+(2+(1+))), 28-(26-(25-)) | **0** | **0** | **0** | **0** | **0** | **0** | 0 | 0 | **0** | **0** | **0** | 0 | 0 | 0 | 0 | 0 | **0** | 0 | 0 | 0 | 0 | **0** | **0** | **0** | 0 | 0 | 0 | 0 |
| 5-(2+(1+)), 50+(1+, 28+(25+)) | **0** | **0** | **0** | **0** | **0** | **0** | 0 | 0 | **0** | **0** | **0** | 0 | 0 | 0 | 0 | 0 | **0** | 0 | 0 | 0 | 0 | **0** | **0** | **0** | 0 | 0 | 0 | 0 |
| 8+(7+), 50+(1+, 28+(26-(25-))) | **0** | **0** | **0** | **0** | **0** | **1** | 0 | 0 | **0** | **0** | **0** | 0 | 0 | 0 | 0 | 0 | **0** | 0 | 0 | 0 | 0 | **0** | **0** | **0** | 0 | 0 | 0 | 0 |
| 6-(5+(2+(1+))), 50+(1+, 8+(7+)) | **0** | **0** | **0** | **1** | **0** | **0** | 0 | 0 | **0** | **0** | **0** | 0 | 0 | 0 | 0 | 0 | **0** | 0 | 0 | 0 | 0 | **0** | **0** | **0** | 0 | 0 | 0 | 0 |
| 6-(5+(2+(1+))), 50+(1+, 28+(25+)) | **0** | **0** | **0** | **0** | **0** | **0** | 0 | 0 | **0** | **0** | **0** | 0 | 0 | 0 | 0 | 0 | **0** | 0 | 0 | 0 | 0 | **0** | **0** | **0** | 0 | 0 | 0 | 0 |
| 5-(2+(1+)), 50+(1+, 28+(26-(25-))) | **0** | **0** | **0** | **0** | **0** | **0** | 0 | 0 | **0** | **0** | **0** | 0 | 0 | 0 | 0 | 0 | **0** | 0 | 0 | 0 | 0 | **0** | **0** | **0** | 0 | 0 | 0 | 1 |
| 6-(5+(2+(1+))), 50+(1+, 28+(26-(25-))) | **0** | **0** | **0** | **0** | **0** | **0** | 0 | 0 | **0** | **0** | **0** | 0 | 0 | 0 | 0 | 0 | **0** | 0 | 0 | 0 | 0 | **0** | **0** | **0** | 0 | 0 | 0 | 0 |

For Hu, this parameter is marked as uncertain in L&G and as neutralized here. It is shown as neutralized because none of the available paths is realized due to either uncertain states or a parameter in the dependency being set to the non-target value.

Parameter 57: *± Feature Spread on Possessives*

| 4 Setability Paths | **It** | **Sal** | **Sp** | **Fr** | **Ptg** | **Rum** | **Lat** | **ClG** | **NTG** | **Gri** | **Grk** | **Got** | **OE** | **E** | **D** | **Nor** | **Blg** | **SC** | **Rus** | **Ir** | **Wel** | **Heb** | **Ar** | **Wo** | **Hu** | **Fin** | **Hi** | **Ba** |
| --- | --- | --- | --- | --- | --- | --- | --- | --- | --- | --- | --- | --- | --- | --- | --- | --- | --- | --- | --- | --- | --- | --- | --- | --- | --- | --- | --- | --- |
| 34+ | **1** | **1** | **1** | **1** | **1** | **1** | **1** | **1** | **1** | **1** | **1** | **1** | **1** | 0 | **0** | **1** | **1** | **1** | **1** | **1** | 0 | **1** | **1** | 1 | **1** | **1** | **1** | 1 |
| 33+(32+) | **1** | **1** | **1** | **1** | **1** | **1** | **1** | **1** | **1** | **1** | **1** | **1** | **1** | 0 | **1** | **1** | **1** | **1** | **1** | **1** | 0 | **1** | **1** | 0 | **0** | **1** | **1** | 0 |
| 35+(6+(5+(2+(1+))), 34+) | **1** | **1** | **1** | **0** | **1** | **1** | **1** | **1** | **1** | **1** | **1** | **1** | **1** | 0 | **0** | **1** | **1** | **1** | **1** | **1** | 0 | **1** | **1** | 0 | **1** | **1** | **1** | 0 |
| 35+(6+(5+(2+(1+))), 33+(32+)) | **1** | **1** | **1** | **0** | **1** | **1** | **1** | **1** | **1** | **1** | **1** | **1** | **1** | 0 | **1** | **1** | **1** | **1** | **1** | **1** | 0 | **1** | **1** | 0 | **0** | **1** | **1** | 0 |

For Wel, this parameter is marked as uncertain in L&G and as neutralized here. It is shown as neutralized because none of the paths is realized due to uncertain states in the dependency.

Parameter 58: *±Feature Spread on Postpositional Genitives*

| 16 Setability Paths | **It** | **Sal** | **Sp** | **Fr** | **Ptg** | **Rum** | **Lat** | **ClG** | **NTG** | **Gri** | **Grk** | **Got** | **OE** | **E** | **D** | **Nor** | **Blg** | **SC** | **Rus** | **Ir** | **Wel** | **Heb** | **Ar** | **Wo** | **Hu** | **Fin** | **Hi** | **Ba** |
| --- | --- | --- | --- | --- | --- | --- | --- | --- | --- | --- | --- | --- | --- | --- | --- | --- | --- | --- | --- | --- | --- | --- | --- | --- | --- | --- | --- | --- |
| 57+(34+), 29+(28+(25+)) | 0 | 0 | 0 | 0 | 0 | 0 | 0 | 0 | 0 | 0 | 0 | 0 | 0 | 0 | 0 | **1** | 0 | 0 | 0 | 0 | 0 | 0 | 0 | 0 | 0 | 0 | **0** | 0 |
| 57+(34+), 29+(27+(25+)) | 0 | 0 | 0 | 0 | 0 | 0 | 0 | 0 | 0 | 0 | 0 | 0 | 0 | 0 | 0 | **0** | 0 | 0 | 0 | 0 | 0 | 0 | 0 | 0 | 0 | 0 | **0** | 0 |
| 57+(34+), 29+(27+(26-(25-))) | 0 | 0 | 0 | 0 | 0 | 0 | 0 | 0 | 0 | 0 | 0 | 0 | 0 | 0 | 0 | **0** | 0 | 0 | 0 | 0 | 0 | 0 | 0 | 0 | 0 | 0 | **1** | 0 |
| 57+(34+), 29+(28+(26-(25-))) | 0 | 0 | 0 | 0 | 0 | 0 | 0 | 0 | 0 | 0 | 0 | 0 | 0 | 0 | 0 | **0** | 0 | 0 | 0 | 0 | 0 | 0 | 0 | 0 | 0 | 0 | **1** | 0 |
| 57+(33+(32+)), 29+(27+(25+)) | 0 | 0 | 0 | 0 | 0 | 0 | 0 | 0 | 0 | 0 | 0 | 0 | 0 | 0 | 0 | **0** | 0 | 0 | 0 | 0 | 0 | 0 | 0 | 0 | 0 | 0 | **0** | 0 |
| 57+(33+(32+)), 29+(28+(25+)) | 0 | 0 | 0 | 0 | 0 | 0 | 0 | 0 | 0 | 0 | 0 | 0 | 0 | 0 | 0 | **1** | 0 | 0 | 0 | 0 | 0 | 0 | 0 | 0 | 0 | 0 | **0** | 0 |
| 57+(33+(32+)), 29+(27+(26-(25-))) | 0 | 0 | 0 | 0 | 0 | 0 | 0 | 0 | 0 | 0 | 0 | 0 | 0 | 0 | 0 | **0** | 0 | 0 | 0 | 0 | 0 | 0 | 0 | 0 | 0 | 0 | **1** | 0 |
| 57+(33+(32+)), 29+(28+(26-(25-))) | 0 | 0 | 0 | 0 | 0 | 0 | 0 | 0 | 0 | 0 | 0 | 0 | 0 | 0 | 0 | **0** | 0 | 0 | 0 | 0 | 0 | 0 | 0 | 0 | 0 | 0 | **1** | 0 |
| 57+(35+(6+(5+(2+(1+)))), 34+), 29+(27+(25+)) | 0 | 0 | 0 | 0 | 0 | 0 | 0 | 0 | 0 | 0 | 0 | 0 | 0 | 0 | 0 | **0** | 0 | 0 | 0 | 0 | 0 | 0 | 0 | 0 | 0 | 0 | **0** | 0 |
| 57+(35+(6+(5+(2+(1+)))), 34+), 29+(28+(25+)) | 0 | 0 | 0 | 0 | 0 | 0 | 0 | 0 | 0 | 0 | 0 | 0 | 0 | 0 | 0 | **1** | 0 | 0 | 0 | 0 | 0 | 0 | 0 | 0 | 0 | 0 | **0** | 0 |
| 57+(35+(6+(5+(2+(1+)))), 34+), 29+(27+(26-(25-))) | 0 | 0 | 0 | 0 | 0 | 0 | 0 | 0 | 0 | 0 | 0 | 0 | 0 | 0 | 0 | **0** | 0 | 0 | 0 | 0 | 0 | 0 | 0 | 0 | 0 | 0 | **1** | 0 |
| 57+(35+(6+(5+(2+(1+)))), 34+), 29+(28+(26-(25-))) | 0 | 0 | 0 | 0 | 0 | 0 | 0 | 0 | 0 | 0 | 0 | 0 | 0 | 0 | 0 | **0** | 0 | 0 | 0 | 0 | 0 | 0 | 0 | 0 | 0 | 0 | **1** | 0 |
| 57+(35+(6+(5+(2+(1+)))), 33+(32+)), 29+(27+(25+)) | 0 | 0 | 0 | 0 | 0 | 0 | 0 | 0 | 0 | 0 | 0 | 0 | 0 | 0 | 0 | **0** | 0 | 0 | 0 | 0 | 0 | 0 | 0 | 0 | 0 | 0 | **0** | 0 |
| 57+(35+(6+(5+(2+(1+)))), 33+(32+)), 29+(28+(25+)) | 0 | 0 | 0 | 0 | 0 | 0 | 0 | 0 | 0 | 0 | 0 | 0 | 0 | 0 | 0 | **1** | 0 | 0 | 0 | 0 | 0 | 0 | 0 | 0 | 0 | 0 | **0** | 0 |
| 57+(35+(6+(5+(2+(1+)))), 33+(32+)), 29+(27+(26-(25-))) | 0 | 0 | 0 | 0 | 0 | 0 | 0 | 0 | 0 | 0 | 0 | 0 | 0 | 0 | 0 | **0** | 0 | 0 | 0 | 0 | 0 | 0 | 0 | 0 | 0 | 0 | **1** | 0 |
| 57+(35+(6+(5+(2+(1+)))), 33+(32+)), 29+(28+(26-(25-))) | 0 | 0 | 0 | 0 | 0 | 0 | 0 | 0 | 0 | 0 | 0 | 0 | 0 | 0 | 0 | **0** | 0 | 0 | 0 | 0 | 0 | 0 | 0 | 0 | 0 | 0 | **1** | 0 |

Parameter 60: *±Consistency Principle*

| 23 Setability Paths | **It** | **Sal** | **Sp** | **Fr** | **Ptg** | **Rum** | **Lat** | **ClG** | **NTG** | **Gri** | **Grk** | **Got** | **OE** | **E** | **D** | **Nor** | **Blg** | **SC** | **Rus** | **Ir** | **Wel** | **Heb** | **Ar** | **Wo** | **Hu** | **Fin** | **Hi** | **Ba** |
| --- | --- | --- | --- | --- | --- | --- | --- | --- | --- | --- | --- | --- | --- | --- | --- | --- | --- | --- | --- | --- | --- | --- | --- | --- | --- | --- | --- | --- |
| 51+ | **0** | 0 | **0** | **0** | **0** | **0** | 0 | 0 | **0** | **0** | 0 | 0 | 0 | 0 | 0 | 0 | 0 | **0** | **0** | 0 | 0 | 0 | 0 | 1 | **0** | 0 | 0 | 1 |
| 43-(42-) | **0** | 0 | **0** | **0** | **0** | **0** | 0 | 0 | **0** | **0** | 0 | 0 | 0 | 1 | 0 | 1 | 0 | **0** | **0** | 0 | 0 | 0 | 0 | 0 | **0** | 0 | 0 | 0 |
| 44-(30-, 43+(42-), 26+(25-)) | **0** | 0 | **0** | **0** | **0** | **0** | 1 | 1 | **0** | **0** | 0 | 0 | 0 | 0 | 0 | 0 | 0 | **0** | **0** | 0 | 0 | 0 | 0 | 0 | **0** | 0 | 0 | 0 |
| 44-(30-, 43+(42-), 27+(25+)) | **0** | 0 | **0** | **0** | **0** | **0** | 0 | 0 | **0** | **0** | 0 | 0 | 0 | 0 | 0 | 0 | 0 | **0** | **0** | 0 | 0 | 0 | 0 | 0 | **0** | 0 | 0 | 0 |
| 45-(32+, 43+(42-), 26-(25-)) | **0** | 0 | **0** | **0** | **0** | **0** | 0 | 0 | **1** | **0** | 1 | 1 | 1 | 0 | 0 | 0 | 0 | **1** | **1** | 0 | 0 | 0 | 0 | 0 | **1** | 0 | 1 | 0 |
| 45-(32+, 43+(42-), 27-(25+)) | **0** | 0 | **0** | **0** | **0** | **0** | 0 | 0 | **0** | **0** | 0 | 0 | 0 | 0 | 0 | 0 | 1 | **0** | **0** | 0 | 0 | 0 | 0 | 0 | **0** | 0 | 0 | 0 |
| 44-(30-, 43+(42-), 27+(26-(25-))) | **0** | 0 | **0** | **0** | **0** | **0** | 0 | 0 | **0** | **0** | 0 | 0 | 0 | 0 | 0 | 0 | 0 | **0** | **0** | 0 | 0 | 0 | 0 | 0 | **0** | 1 | 0 | 0 |
| 45-(32+, 43+(42-), 27-(26-(25-))) | **0** | 0 | **0** | **0** | **0** | **0** | 0 | 0 | **0** | **0** | 0 | 0 | 0 | 0 | 0 | 0 | 0 | **0** | **0** | 0 | 0 | 0 | 0 | 0 | **1** | 0 | 0 | 0 |
| 46+(45+(32+, 43+(42-), 26-(25-))) | **0** | 0 | **0** | **0** | **0** | **1** | 0 | 0 | **0** | **1** | 0 | 0 | 0 | 0 | 0 | 0 | 0 | **0** | **0** | 0 | 0 | 0 | 0 | 0 | **0** | 0 | 0 | 0 |
| 46+(45+(32+, 43+(42-), 27-(25+))) | **1** | 1 | **1** | **1** | **1** | **0** | 0 | 0 | **0** | **0** | 0 | 0 | 0 | 0 | 0 | 0 | 0 | **0** | **0** | 0 | 0 | 0 | 0 | 0 | **0** | 0 | 0 | 0 |
| 45-(32+, 44+(30-, 43+(42-), 26+(25-))) | **0** | 0 | **0** | **0** | **0** | **0** | 0 | 0 | **0** | **0** | 0 | 0 | 0 | 0 | 0 | 0 | 0 | **0** | **0** | 0 | 0 | 0 | 0 | 0 | **0** | 0 | 0 | 0 |
| 45-(32+, 44+(30-, 43+(42-), 27+(25+))) | **0** | 0 | **0** | **0** | **0** | **0** | 0 | 0 | **0** | **0** | 0 | 0 | 0 | 0 | 1 | 0 | 0 | **0** | **0** | 0 | 0 | 0 | 0 | 0 | **0** | 0 | 0 | 0 |
| 46+(45+(32+, 43+(42-), 27-(26-(25-)))) | **0** | 0 | **0** | **0** | **0** | **1** | 0 | 0 | **0** | **0** | 0 | 0 | 0 | 0 | 0 | 0 | 0 | **0** | **0** | 0 | 0 | 0 | 0 | 0 | **0** | 0 | 0 | 0 |
| 47-(46+(45+(32+, 43+(42-), 26-(25-)))) | **0** | 0 | **0** | **0** | **0** | **1** | 0 | 0 | **0** | **0** | 0 | 0 | 0 | 0 | 0 | 0 | 0 | **0** | **0** | 0 | 0 | 0 | 0 | 0 | **0** | 0 | 0 | 0 |
| 47-(46+(45+(32+, 43+(42-), 27-(25+)))) | **1** | 0 | **1** | **1** | **1** | **0** | 0 | 0 | **0** | **0** | 0 | 0 | 0 | 0 | 0 | 0 | 0 | **0** | **0** | 0 | 0 | 0 | 0 | 0 | **0** | 0 | 0 | 0 |
| 47-(46+(45+(32+, 43+(42-), 27-(26-(25-))))) | **0** | 0 | **0** | **0** | **0** | **1** | 0 | 0 | **0** | **0** | 0 | 0 | 0 | 0 | 0 | 0 | 0 | **0** | **0** | 0 | 0 | 0 | 0 | 0 | **0** | 0 | 0 | 0 |
| 45-(32+, 44+(30-, 43+(42-), 27+(26-(25-)))) | **0** | 0 | **0** | **0** | **0** | **0** | 0 | 0 | **1** | **0** | 1 | 1 | 1 | 0 | 0 | 0 | 0 | **1** | **1** | 0 | 0 | 0 | 0 | 0 | **0** | 0 | 0 | 0 |
| 46+(45+(32+, 44+(30-, 43+(42-), 26+(25-)))) | **0** | 0 | **0** | **0** | **0** | **0** | 0 | 0 | **0** | **0** | 0 | 0 | 0 | 0 | 0 | 0 | 0 | **0** | **0** | 0 | 0 | 0 | 0 | 0 | **0** | 0 | 0 | 0 |
| 46+(45+(32+, 44+(30-, 43+(42-), 27+(25+)))) | **0** | 0 | **0** | **0** | **0** | **0** | 0 | 0 | **0** | **0** | 0 | 0 | 0 | 0 | 0 | 0 | 0 | **0** | **0** | 1 | 1 | 0 | 0 | 0 | **0** | 0 | 0 | 0 |
| 46+(45+(32+, 44+(30-, 43+(42-), 27+(26-(25-))))) | **0** | 0 | **0** | **0** | **0** | **0** | 0 | 0 | **0** | **1** | 0 | 0 | 0 | 0 | 0 | 0 | 0 | **0** | **0** | 0 | 0 | 0 | 0 | 0 | **0** | 0 | 0 | 0 |
| 47-(46+(45+(32+, 44+(30-, 43+(42-), 26+(25-))))) | **0** | 0 | **0** | **0** | **0** | **0** | 0 | 0 | **0** | **0** | 0 | 0 | 0 | 0 | 0 | 0 | 0 | **0** | **0** | 0 | 0 | 0 | 0 | 0 | **0** | 0 | 0 | 0 |
| 47-(46+(45+(32+, 44+(30-, 43+(42-), 27+(25+))))) | **0** | 0 | **0** | **0** | **0** | **0** | 0 | 0 | **0** | **0** | 0 | 0 | 0 | 0 | 0 | 0 | 0 | **0** | **0** | 0 | 0 | 0 | 0 | 0 | **0** | 0 | 0 | 0 |
| 47-(46+(45+(32+, 44+(30-, 43+(42-), 27+(26-(25-)))))) | **0** | 0 | **0** | **0** | **0** | **0** | 0 | 0 | **0** | **0** | 0 | 0 | 0 | 0 | 0 | 0 | 0 | **0** | **0** | 0 | 0 | 0 | 0 | 0 | **0** | 0 | 0 | 0 |

For Sal, Ir, Wel, and Hi, this parameter is marked as neutralized in L&G and as settable in all four cases here. It is settable in all four cases, three out of four on different paths.

Parameter 61: *±Null N-Licensing Article*

| 12 Setability Paths | **It** | **Sal** | **Sp** | **Fr** | **Ptg** | **Rum** | **Lat** | **ClG** | **NTG** | **Gri** | **Grk** | **Got** | **OE** | **E** | **D** | **Nor** | **Blg** | **SC** | **Rus** | **Ir** | **Wel** | **Heb** | **Ar** | **Wo** | **Hu** | **Fin** | **Hi** | **Ba** |
| --- | --- | --- | --- | --- | --- | --- | --- | --- | --- | --- | --- | --- | --- | --- | --- | --- | --- | --- | --- | --- | --- | --- | --- | --- | --- | --- | --- | --- |
| 12-(7+), 51+ | 0 | 0 | 0 | **0** | 0 | 0 | 0 | 0 | 0 | 0 | 0 | 0 | 0 | 0 | 0 | 0 | 0 | 0 | 0 | 0 | 0 | **0** | **0** | 1 | 0 | 0 | 0 | **0** |
| 5-(2+(1+)), 51+ | 0 | 0 | 0 | **0** | 0 | 0 | 0 | 0 | 0 | 0 | 0 | 0 | 0 | 0 | 0 | 0 | 0 | 0 | 0 | 0 | 0 | **0** | **0** | 1 | 0 | 0 | 0 | **1** |
| 6-(5+(2+(1+))), 51+ | 0 | 0 | 0 | **0** | 0 | 0 | 0 | 0 | 0 | 0 | 0 | 0 | 0 | 0 | 0 | 0 | 0 | 0 | 0 | 0 | 0 | **0** | **0** | 0 | 0 | 0 | 0 | **0** |
| 12-(7+), 50+(1+, 8+(7+)) | 1 | 1 | 1 | **1** | 1 | 0 | 0 | 1 | 1 | 1 | 1 | 0 | 0 | 0 | 0 | 0 | 0 | 0 | 0 | 0 | 0 | **1** | **1** | 0 | 0 | 0 | 0 | **0** |
| 12-(7+), 50+(1+, 28+(25+)) | 0 | 0 | 0 | **0** | 0 | 0 | 0 | 0 | 0 | 0 | 0 | 0 | 0 | 0 | 0 | 0 | 0 | 0 | 0 | 0 | 0 | **1** | **1** | 0 | 0 | 0 | 0 | **0** |
| 5-(2+(1+)), 50+(1+, 8+(7+)) | 0 | 0 | 0 | **0** | 0 | 0 | 0 | 0 | 0 | 0 | 0 | 0 | 0 | 0 | 0 | 0 | 0 | 0 | 0 | 0 | 0 | **0** | **0** | 0 | 0 | 0 | 0 | **0** |
| 5-(2+(1+)), 50+(1+, 28+(25+)) | 0 | 0 | 0 | **0** | 0 | 0 | 0 | 0 | 0 | 0 | 0 | 0 | 0 | 0 | 0 | 0 | 0 | 0 | 0 | 0 | 0 | **0** | **0** | 0 | 0 | 0 | 0 | **0** |
| 6-(5+(2+(1+))), 50+(1+, 8+(7+)) | 0 | 0 | 0 | **1** | 0 | 0 | 0 | 0 | 0 | 0 | 0 | 0 | 0 | 0 | 0 | 0 | 0 | 0 | 0 | 0 | 0 | **0** | **0** | 0 | 0 | 0 | 0 | **0** |
| 12-(7+), 50+(1+, 28+(26-(25-))) | 0 | 0 | 0 | **0** | 0 | 0 | 0 | 0 | 0 | 0 | 0 | 0 | 0 | 0 | 0 | 0 | 0 | 0 | 0 | 0 | 0 | **0** | **0** | 0 | 0 | 0 | 0 | **0** |
| 6-(5+(2+(1+))), 50+(1+, 28+(25+)) | 0 | 0 | 0 | **0** | 0 | 0 | 0 | 0 | 0 | 0 | 0 | 0 | 0 | 0 | 0 | 0 | 0 | 0 | 0 | 0 | 0 | **0** | **0** | 0 | 0 | 0 | 0 | **0** |
| 5-(2+(1+)), 50+(1+, 28+(26-(25-))) | 0 | 0 | 0 | **0** | 0 | 0 | 0 | 0 | 0 | 0 | 0 | 0 | 0 | 0 | 0 | 0 | 0 | 0 | 0 | 0 | 0 | **0** | **0** | 0 | 0 | 0 | 0 | **1** |
| 6-(5+(2+(1+))), 50+(1+, 28+(26-(25-))) | 0 | 0 | 0 | **0** | 0 | 0 | 0 | 0 | 0 | 0 | 0 | 0 | 0 | 0 | 0 | 0 | 0 | 0 | 0 | 0 | 0 | **0** | **0** | 0 | 0 | 0 | 0 | **0** |

For Hu, this parameter is marked as uncertain in L&G and as neutralized here. It is shown as neutralized because none of the paths is realized due to either uncertain states or a parameter in the dependency being set to the non-target value.

Parameter 62: *±Grammaticalized Geographical Article*

| 36 Setability Paths | **It** | **Sal** | **Sp** | **Fr** | **Ptg** | **Rum** | **Lat** | **ClG** | **NTG** | **Gri** | **Grk** | **Got** | **OE** | **E** | **D** | **Nor** | **Blg** | **SC** | **Rus** | **Ir** | **Wel** | **Heb** | **Ar** | **Wo** | **Hu** | **Fin** | **Hi** | **Ba** |
| --- | --- | --- | --- | --- | --- | --- | --- | --- | --- | --- | --- | --- | --- | --- | --- | --- | --- | --- | --- | --- | --- | --- | --- | --- | --- | --- | --- | --- |
| 7+, 22-(21-, 7+) | 0 | 0 | 0 | **0** | 0 | **0** | 0 | 1 | 1 | **0** | 0 | 0 | 0 | 0 | 0 | 0 | 0 | 0 | 0 | **1** | **1** | 1 | 1 | 0 | 0 | 0 | 0 | 0 |
| 7+, 23-(22+(21-, 7+)) | 0 | 0 | 0 | **0** | 0 | **0** | 0 | 0 | 0 | **0** | 0 | 0 | 1 | 0 | 0 | 0 | 1 | 0 | 0 | **0** | **0** | 0 | 0 | 0 | 0 | 0 | 0 | 0 |
| 5-(2+(1+)), 22-(21-, 7+) | 0 | 0 | 0 | **0** | 0 | **0** | 0 | 0 | 0 | **0** | 0 | 0 | 0 | 0 | 0 | 0 | 0 | 0 | 0 | **0** | **0** | 0 | 0 | 0 | 0 | 0 | 0 | 0 |
| 7+, 22-(21-, 5-(2+(1+))) | 0 | 0 | 0 | **0** | 0 | **0** | 0 | 0 | 0 | **0** | 0 | 0 | 0 | 0 | 0 | 0 | 0 | 0 | 0 | **0** | **0** | 0 | 0 | 0 | 0 | 0 | 0 | 0 |
| 6-(5+(2+(1+))), 22-(21-, 7+) | 0 | 0 | 0 | **0** | 0 | **0** | 0 | 0 | 0 | **0** | 0 | 0 | 0 | 0 | 0 | 0 | 0 | 0 | 0 | **0** | **0** | 0 | 0 | 0 | 0 | 0 | 0 | 0 |
| 7+, 22-(21-, 6-(5+(2+(1+)))) | 0 | 0 | 0 | **0** | 0 | **0** | 0 | 0 | 0 | **0** | 0 | 0 | 0 | 0 | 0 | 0 | 0 | 0 | 0 | **0** | **0** | 0 | 0 | 0 | 0 | 0 | 0 | 0 |
| 5-(2+(1+)), 23-(22+(21-, 7+)) | 0 | 0 | 0 | **0** | 0 | **0** | 0 | 0 | 0 | **0** | 0 | 0 | 0 | 0 | 0 | 0 | 0 | 0 | 0 | **0** | **0** | 0 | 0 | 0 | 0 | 0 | 0 | 0 |
| 7+, 23-(22+(21-, 5-(2+(1+)))) | 0 | 0 | 0 | **0** | 0 | **0** | 0 | 0 | 0 | **0** | 0 | 0 | 0 | 0 | 0 | 0 | 0 | 0 | 0 | **0** | **0** | 0 | 0 | 0 | 0 | 0 | 0 | 0 |
| 7+, 45+(32+, 43+(42-), 26-(25-)) | 0 | 0 | 0 | **0** | 0 | **1** | 0 | 0 | 0 | **1** | 0 | 0 | 0 | 0 | 0 | 0 | 0 | 0 | 0 | **0** | **0** | 0 | 0 | 0 | 0 | 0 | 0 | 0 |
| 7+, 45+(32+, 43+(42-), 27-(25+)) | 1 | 1 | 1 | **1** | 1 | **0** | 0 | 0 | 0 | **0** | 0 | 0 | 0 | 0 | 0 | 0 | 0 | 0 | 0 | **0** | **0** | 0 | 0 | 0 | 0 | 0 | 0 | 0 |
| 5-(2+(1+)), 22-(21-, 5-(2+(1+))) | 0 | 0 | 0 | **0** | 0 | **0** | 0 | 0 | 0 | **0** | 0 | 0 | 0 | 0 | 0 | 0 | 0 | 0 | 0 | **0** | **0** | 0 | 0 | 0 | 0 | 0 | 0 | 0 |
| 6-(5+(2+(1+))), 23-(22+(21-, 7+)) | 0 | 0 | 0 | **0** | 0 | **0** | 0 | 0 | 0 | **0** | 0 | 0 | 0 | 0 | 0 | 0 | 0 | 0 | 0 | **0** | **0** | 0 | 0 | 0 | 0 | 0 | 0 | 0 |
| 7+, 23-(22+(21-, 6-(5+(2+(1+))))) | 0 | 0 | 0 | **0** | 0 | **0** | 0 | 0 | 0 | **0** | 0 | 0 | 0 | 0 | 0 | 0 | 0 | 0 | 0 | **0** | **0** | 0 | 0 | 0 | 0 | 0 | 0 | 0 |
| 7+, 45+(32+, 43+(42-), 27-(26-(25-))) | 0 | 0 | 0 | **0** | 0 | **1** | 0 | 0 | 0 | **0** | 0 | 0 | 0 | 0 | 0 | 0 | 0 | 0 | 0 | **0** | **0** | 0 | 0 | 0 | 0 | 0 | 0 | 0 |
| 5-(2+(1+)), 22-(21-, 6-(5+(2+(1+)))) *predicted but impossible, mutually exclusive values of 5 | 0 | 0 | 0 | **0** | 0 | **0** | 0 | 0 | 0 | **0** | 0 | 0 | 0 | 0 | 0 | 0 | 0 | 0 | 0 | **0** | **0** | 0 | 0 | 0 | 0 | 0 | 0 | 0 |
| 6-(5+(2+(1+))), 22-(21-, 5-(2+(1+))) *predicted but impossible, mutually exclusive values of 5 | 0 | 0 | 0 | **0** | 0 | **0** | 0 | 0 | 0 | **0** | 0 | 0 | 0 | 0 | 0 | 0 | 0 | 0 | 0 | **0** | **0** | 0 | 0 | 0 | 0 | 0 | 0 | 0 |
| 5-(2+(1+)), 23-(22+(21-, 5-(2+(1+)))) | 0 | 0 | 0 | **0** | 0 | **0** | 0 | 0 | 0 | **0** | 0 | 0 | 0 | 0 | 0 | 0 | 0 | 0 | 0 | **0** | **0** | 0 | 0 | 0 | 0 | 0 | 0 | 1 |
| 6-(5+(2+(1+))), 22-(21-, 6-(5+(2+(1+)))) | 0 | 0 | 0 | **0** | 0 | **0** | 0 | 0 | 0 | **0** | 0 | 0 | 0 | 0 | 0 | 0 | 0 | 0 | 0 | **0** | **0** | 0 | 0 | 0 | 0 | 0 | 0 | 0 |
| 5-(2+(1+)), 45+(32+, 43+(42-), 26-(25-)) | 0 | 0 | 0 | **0** | 0 | **0** | 0 | 0 | 0 | **0** | 0 | 0 | 0 | 0 | 0 | 0 | 0 | 0 | 0 | **0** | **0** | 0 | 0 | 0 | 0 | 0 | 0 | 0 |
| 5-(2+(1+)), 45+(32+, 43+(42-), 27-(25+)) | 0 | 0 | 0 | **0** | 0 | **0** | 0 | 0 | 0 | **0** | 0 | 0 | 0 | 0 | 0 | 0 | 0 | 0 | 0 | **0** | **0** | 0 | 0 | 0 | 0 | 0 | 0 | 0 |
| 7+, 45+(32+, 44+(30-, 43+(42-), 26+(25-))) | 0 | 0 | 0 | **0** | 0 | **0** | 0 | 0 | 0 | **0** | 0 | 0 | 0 | 0 | 0 | 0 | 0 | 0 | 0 | **0** | **0** | 0 | 0 | 0 | 0 | 0 | 0 | 0 |
| 7+, 45+(32+, 44+(30-, 43+(42-), 27+(25+))) | 0 | 0 | 0 | **0** | 0 | **0** | 0 | 0 | 0 | **0** | 0 | 0 | 0 | 0 | 0 | 0 | 0 | 0 | 0 | **1** | **1** | 0 | 0 | 0 | 0 | 0 | 0 | 0 |
| 5-(2+(1+)), 23-(22+(21-, 6-(5+(2+(1+))))) *predicted but impossible, mutually exclusive values of 5 | 0 | 0 | 0 | **0** | 0 | **0** | 0 | 0 | 0 | **0** | 0 | 0 | 0 | 0 | 0 | 0 | 0 | 0 | 0 | **0** | **0** | 0 | 0 | 0 | 0 | 0 | 0 | 0 |
| 6-(5+(2+(1+))), 23-(22+(21-, 5-(2+(1+)))) *predicted but impossible, mutually exclusive values of 5 | 0 | 0 | 0 | **0** | 0 | **0** | 0 | 0 | 0 | **0** | 0 | 0 | 0 | 0 | 0 | 0 | 0 | 0 | 0 | **0** | **0** | 0 | 0 | 0 | 0 | 0 | 0 | 0 |
| 5-(2+(1+)), 45+(32+, 43+(42-), 27-(26-(25-))) | 0 | 0 | 0 | **0** | 0 | **0** | 0 | 0 | 0 | **0** | 0 | 0 | 0 | 0 | 0 | 0 | 0 | 0 | 0 | **0** | **0** | 0 | 0 | 0 | 0 | 0 | 0 | 0 |
| 6-(5+(2+(1+))), 45+(32+, 43+(42-), 26-(25-)) | 0 | 0 | 0 | **0** | 0 | **0** | 0 | 0 | 0 | **0** | 0 | 0 | 0 | 0 | 0 | 0 | 0 | 0 | 0 | **0** | **0** | 0 | 0 | 0 | 0 | 0 | 0 | 0 |
| 6-(5+(2+(1+))), 45+(32+, 43+(42-), 27-(25+)) | 0 | 0 | 0 | **1** | 0 | **0** | 0 | 0 | 0 | **0** | 0 | 0 | 0 | 0 | 0 | 0 | 0 | 0 | 0 | **0** | **0** | 0 | 0 | 0 | 0 | 0 | 0 | 0 |
| 7+, 45+(32+, 44+(30-, 43+(42-), 27+(26-(25-)))) | 0 | 0 | 0 | **0** | 0 | **0** | 0 | 0 | 0 | **1** | 0 | 0 | 0 | 0 | 0 | 0 | 0 | 0 | 0 | **0** | **0** | 0 | 0 | 0 | 0 | 0 | 0 | 0 |
| 6-(5+(2+(1+))), 23-(22+(21-, 6-(5+(2+(1+))))) | 0 | 0 | 0 | **0** | 0 | **0** | 0 | 0 | 0 | **0** | 0 | 0 | 0 | 0 | 0 | 0 | 0 | 0 | 0 | **0** | **0** | 0 | 0 | 0 | 0 | 0 | 0 | 0 |
| 6-(5+(2+(1+))), 45+(32+, 43+(42-), 27-(26-(25-))) | 0 | 0 | 0 | **0** | 0 | **0** | 0 | 0 | 0 | **0** | 0 | 0 | 0 | 0 | 0 | 0 | 0 | 0 | 0 | **0** | **0** | 0 | 0 | 0 | 0 | 0 | 0 | 0 |
| 5-(2+(1+)), 45+(32+, 44+(30-, 43+(42-), 26+(25-))) | 0 | 0 | 0 | **0** | 0 | **0** | 0 | 0 | 0 | **0** | 0 | 0 | 0 | 0 | 0 | 0 | 0 | 0 | 0 | **0** | **0** | 0 | 0 | 0 | 0 | 0 | 0 | 0 |
| 5-(2+(1+)), 45+(32+, 44+(30-, 43+(42-), 27+(25+))) | 0 | 0 | 0 | **0** | 0 | **0** | 0 | 0 | 0 | **0** | 0 | 0 | 0 | 0 | 0 | 0 | 0 | 0 | 0 | **0** | **0** | 0 | 0 | 0 | 0 | 0 | 0 | 0 |
| 6-(5+(2+(1+))), 45+(32+, 44+(30-, 43+(42-), 26+(25-))) | 0 | 0 | 0 | **0** | 0 | **0** | 0 | 0 | 0 | **0** | 0 | 0 | 0 | 0 | 0 | 0 | 0 | 0 | 0 | **0** | **0** | 0 | 0 | 0 | 0 | 0 | 0 | 0 |
| 6-(5+(2+(1+))), 45+(32+, 44+(30-, 43+(42-), 27+(25+))) | 0 | 0 | 0 | **0** | 0 | **0** | 0 | 0 | 0 | **0** | 0 | 0 | 0 | 0 | 0 | 0 | 0 | 0 | 0 | **0** | **0** | 0 | 0 | 0 | 0 | 0 | 0 | 0 |
| 5-(2+(1+)), 45+(32+, 44+(30-, 43+(42-), 27+(26-(25-)))) | 0 | 0 | 0 | **0** | 0 | **0** | 0 | 0 | 0 | **0** | 0 | 0 | 0 | 0 | 0 | 0 | 0 | 0 | 0 | **0** | **0** | 0 | 0 | 0 | 0 | 0 | 0 | 0 |
| 6-(5+(2+(1+))), 45+(32+, 44+(30-, 43+(42-), 27+(26-(25-)))) | 0 | 0 | 0 | **0** | 0 | **0** | 0 | 0 | 0 | **0** | 0 | 0 | 0 | 0 | 0 | 0 | 0 | 0 | 0 | **0** | **0** | 0 | 0 | 0 | 0 | 0 | 0 | 0 |

For Hu, this parameter is marked as set to ‘-’ in L&G and as neutralized here. It is neutralized because none of the paths is realizable.

**Appendix S3: Code**

package Language_Analyser;

import java.util.HashMap;

import java.util.Iterator;

import java.util.Map;

public class ChildPath

{

// List all the dependencies that comprise this path

private HashMap dependecyTable;

private String LanguageInput[];

// The language input data table

public ChildPath(String langData[])

{

this.dependecyTable = new HashMap();

this.LanguageInput = langData;

}

public boolean ScanChildPath()

{

boolean result = true;

Iterator setList = this.dependecyTable.entrySet().iterator();

while(setList.hasNext())

{

Map.Entry tempEntry = (Map.Entry)setList.next();

String tempKey = (String)tempEntry.getKey();

// Search the language parameter state

String LangVal = this.searchLangData(tempKey);

// If the state does not equal the target symbol

// The whole path is unsettable so break the loop and return false

//System.out.println("Comparing <"+tempEntry.getValue()+","+LangVal+">");

if (!tempEntry.getValue().equals(LangVal))

{

result=false;

break;

}

}

//System.out.println("Final result:"+result);

return result;

}

// Searches in the table that was created by the input Language File

private String searchLangData(String i)

{

int index = Integer.parseInt(i);

return this.LanguageInput[index-1];

}

// All the dependencies will be stored in HashMap

// We will have pairs of <Key,Value> where Key=parameter_ID && Value=symbol

public void addNewDependency(String parameter_ID,String symbol)

{

this.dependecyTable.put(parameter_ID, symbol);

}

}

package Language_Analyser;

import java.io.*;

public class Language_Analyser{

public static ChildPath[] ParameterPaths;

public static String[] fileInputValues;

public static void main(String[] args) throws IOException

{

File folder = new File("Input_Language_Data"), tmpFile;

File[] listOfFiles = folder.listFiles();

fileInputValues = new String[62];

if(listOfFiles == null )

{

System.out.println("Folder with input data was not found!");

File cwd = new File(".");

System.out.println("Working Directory:" + cwd.getAbsoluteFile());

return;

}

for (int i = 0; i < listOfFiles.length; i++)

{

if (listOfFiles[i].isFile() &&

listOfFiles[i].getName().endsWith(".txt"))

{

tmpFile = listOfFiles[i];

ReadFileContents(tmpFile,fileInputValues);

// =============== EDITABLE CODE SECTION ================ //

// Sample code: Parameter 10

// The parameter has three setability paths so you have to

// specify this in the next line inside the parenthesis

ParameterPaths = Language_Analyser.createPaths(3);

// For each path, define its number and

// subtract 1 each time. In the following lines

// we are going to set the first path so in the

// brackets we specify zero (path_index - 1 = 1 - 1 = 0)

// The realization of each path depends on the status of the relevant input nodes

// For each dependency, specify first the number of the input node(s) and

// then the state(s), as shown in the

// following example

ParameterPaths[0].addNewDependency("7", "+");

// For the second path we have to change the index to 1

// (path_index -1 = 2 - 1 = 1)

ParameterPaths[1].addNewDependency("5", "-");

ParameterPaths[1].addNewDependency("2", "+");

ParameterPaths[1].addNewDependency("1", "+");

// Finally, the index for the third path is 2 (path_index -1 = 3 - 1 = 2)

ParameterPaths[2].addNewDependency("6", "-");

ParameterPaths[2].addNewDependency("5", "+");

ParameterPaths[2].addNewDependency("2", "+");

ParameterPaths[2].addNewDependency("1", "+");

// =========== END OF EDITABLE CODE SECTION ============= //

Language_Analyser.showResults(tmpFile);

}

}

}

public static ChildPath[] createPaths(int numberOfPaths)

{

ChildPath Parameter_Paths[] = null;

if (!(numberOfPaths > 0))
